# Lipids, lipid-modifying drug target genes and migraine: a Mendelian randomization study

**DOI:** 10.1186/s10194-023-01633-x

**Published:** 2023-08-18

**Authors:** Yaodan Bi, Yinchao Zhu, Shuai Tang, Yuguang Huang

**Affiliations:** 1 Department of Anesthesiology, Peking Union Medical College and Chinese Academy of Medical Sciences, Peking Union Medical College Hospital, No.1, Shuaifuyuan, Beijing, China; 2 Department of Anesthesiology, West China Hospital, Sichuan University, No.37, Guoxue Valley, Chengdu, Sichuan China

**Keywords:** Migraine, Lipids, Drug target Mendelian randomization

## Abstract

**Introduction:**

Migraine, a prevalent headache disorder with unclear mechanisms and limited treatments, may be influenced by dyslipidemia and genetic factors. Statins and emerging lipid-modifying agents show potential but lack evidence for migraine management. Mendelian Randomization analysis offers insights into causal relationships and therapeutic targets. This study aims to explore genetically predicted lipid traits, drug targets, and their association with migraine risk.

**Method:**

We conducted Mendelian randomization (MR) analyses utilizing genetic variants associated with lipid traits and variants in genes encoding the protein targets of various classes of lipid-lowering drugs. The specific drug classes investigated included HMGCR, PCSK9, NPC1L1, ABCG5/ABCG8, LDLR, LPL, ANGPTL3, APOB, CETP, and APOC3. To determine the effects on migraine risk, we meta-analyzed MR estimates for regional variants using data from two large sample sets. The genetic variants were weighted based on their associations with specific lipid traits, such as low-density lipoprotein cholesterol (LDL-C), high-density lipoprotein cholesterol (HDL-C), Apolipoprotein A1, and Apolipoprotein B. To obtain association weights, we utilized data from lipid genetics consortia. For lipid-modifying drug targets that exhibited suggestive significance, we further employed expression quantitative trait locus (eQTL) data. Additionally, we performed colocalization analysis to assess genetic confounding.

**Result:**

The use of genetic proxies for HMGCR inhibition demonstrated a significant association with a decreased risk of migraine in the FinnGen dataset (OR = 0.64, 95% CI: 0.46–0.88, *p* = 0.0006) and a nearly significant association in the Choquet dataset (OR = 0.78, 95% CI: 0.60–1.01, *p* = 0.06). When pooling the estimates, the overall effect size showed a reduced risk of migraine (OR = 0.73, 95% CI: 0.60–0.89, *p* = 0.0016). Similarly, genetic mimicry of LPL enhancement was associated with a lower risk of migraine in the FinnGen dataset (OR = 0.82, 95% CI: 0.69–0.96, *p* = 0.01) and the Choquet dataset (OR = 0.91, 95% CI: 0.83–0.99, *p* = 0.03). Pooling the estimates showed a consistent effect size (OR = 0.89, 95% CI: 0.83–0.96, *p* = 0.002). Sensitivity analyses yielded no statistically significant evidence of bias arising from pleiotropy or genetic confounding.

**Conclusion:**

In the study, it was observed that among the 10 lipid-lowering drug targets investigated, LPL and HMGCR showed significant associations with migraine risk. These findings indicate that LPL and HMGCR have the potential to serve as candidate drug targets for the treatment or prevention of migraines.

**Supplementary Information:**

The online version contains supplementary material available at 10.1186/s10194-023-01633-x.

## Introduction

Migraine, a prevalent disorder typified by recurrent unilateral headaches accompanied by concomitant neurological manifestations, manifests as a common affliction within headache disorders [1, 2]. It affects a substantial portion of the population, with approximately 13% of men and 33% of women experiencing migraines at some point in their lives. Among individuals under the age of 50, it ranks as a prominent cause of disability globally [1]. Despite extensive research, the exact pathological mechanism underlying migraine remains poorly understood, posing challenges for the development of more effective treatments. Although various medications are employed for migraine management, their efficacy is not universally established, and their use is often limited due to adverse effects [3, 4]. Furthermore, recurrent symptoms may necessitate increased drug intake, which can lead to excessive use and dissatisfaction with available treatment options.

Several observational studies had put forth several indications pointing to a potential association between dyslipidemia and various aspects of migraine, encompassing its occurrence, frequency, and intensity [5–7]. Furthermore, individuals afflicted by migraines exhibit an elevated susceptibility to both stroke and coronary heart disease (CHD) [8, 9]. Lipid abnormalities may also play a crucial role in linking vascular diseases with migraine [10]. Furthermore, earlier investigations have shed light on the existence of shared genetic factors that underlie blood lipoprotein subfractions and migraine [11, 12]. However, it is crucial to acknowledge that the inherent nature of observational cohort studies renders them vulnerable to residual confounding and the potential for reverse causation, thereby impeding our ability to definitively establish a conclusive causal relationship between dyslipidemia and the risk of migraines.

Statins are recognized as a widely employed class of lipid-modifying drugs recommended for both primary and secondary prevention of ischemic stroke and CHD [13–15]. Despite the existence of few epidemiological investigations and small-scale randomized controlled trials focusing on the utility of statins in the realm of migraine prevention, the precise effects of these medications on migraines remain enigmatic [16, 17]. Additionally, novel lipid-modifying agents like PCSK9 inhibitors have emerged, demonstrating their effectiveness in reducing the risk of vascular diseases. However, their specific impact on migraines has yet to be established.

Mendelian Randomization (MR) analysis provides a valuable alternative to randomized clinical trials by leveraging genetic variations associated with a specific exposure. By employing this approach, it becomes possible to assess causal relationships and identify potential therapeutic targets, subsequently subjecting them to investigation in clinical trials. In the context of this study, a two-sample Mendelian randomization (MR) methodology was employed to examine the potential causal association between genetically predicted lipid traits, lipid-modifying targets, and the occurrence of migraines. Initially, univariable MR analyses were conducted to assess the relationship between genetically predicted lipid traits, encompassing circulating lipids and apolipoproteins, and the risk of migraines. This analysis aimed to establish a direct association between these lipid traits and the occurrence of migraines. Subsequently, multivariable MR analyses were employed to evaluate the mediating effects of common risk factors that are known to be associated with migraines. These risk factors include smoking, alcohol consumption, body mass index (BMI), major depression, and blood pressure. By accounting for these potential mediators, the aim was to investigate whether the observed relationships between genetically predicted lipid traits and migraines could be explained, at least partially, by these risk factors. Finally, MR studies focusing on drug targets were conducted to scrutinize the association between genetically predicted lipid modification and the risk of migraines at various gene targets. This analysis aimed to investigate whether specific gene targets involved in lipid modification could potentially impact the risk of migraines.

## Methods

**Fig. 1:**
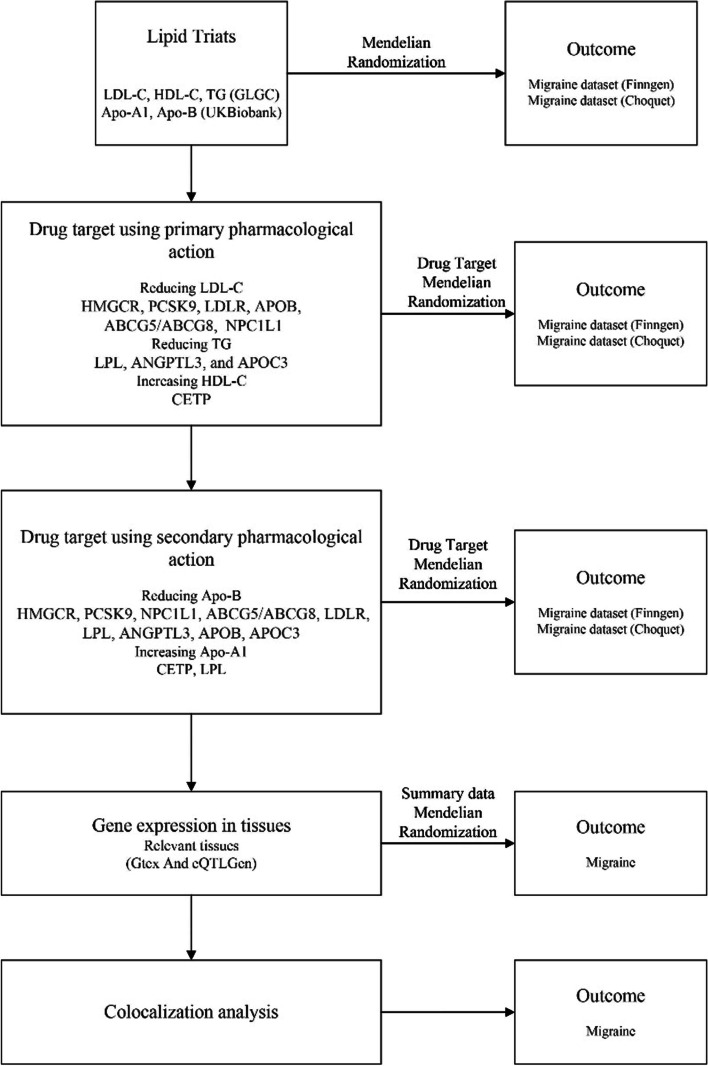
Overview of the study design

This study followed the guidelines outlined in the Strengthening the Reporting of Observational Studies in Epidemiology-Mendelian Randomization (STROBE-MR) reporting guidelines (Table S1) [18]. The study design is visually represented in Fig. 1, offering a comprehensive depiction. To acquire the necessary data, publicly accessible summary-level data originating from genome-wide association studies (GWAS) and expression quantitative trait loci (eQTL) studies were employed. Table S2 presents detailed information regarding these datasets.

### Genetic instrumental variables for lipids and lipid modifying targets

To identify independent instrumental variables associated with lipid traits, we utilized a genome-wide association study (GWAS) performed by the Global Lipids Genetics Consortium (GLGC) [19]. Independent genetic variants associated with LDL-C (low density lipoprotein cholesterol), TG (triglyceride), and HDL-C (high density lipoprotein cholesterol), were selected, meeting a linkage disequilibrium (LD) clumping threshold of r^2 < 0.001 and a physical distance threshold of 10,000 kb. SNPs with missing data and lacking suitable proxies were excluded from the analysis. Additionally, genetic instrumental variants associated with Apo-A1 (Apolipoprotein A1) and Apo-B (Apolipoprotein B) were extracted from another separate GWAS conducted by Nightingale Health, which utilized UK Biobank plasma samples. The same methods were applied [20].

We utilized the DrugBank database to identify the genes encoding with the pharmacological targets of these drugs (https://go.drugbank.com/). We further classified the target genes based on their primary pharmacological action (Table S3). These include the CETP (cholesteryl ester transfer protein) gene as the target gene for increasing HDL-C levels, HMGCR (3-hydroxy-3-methylglutaryl-CoA reductase), LDLR (low density lipoprotein receptor), APOB (apolipoprotein B), ABCG5 (ATP binding cassette subfamily G member 5), and ABCG8 (ATP binding cassette subfamily G member 8), PCSK9 (proprotein convertase subtilisin/kexin type 9), and NPC1L1 (Niemann-Pick C1 like intracellular cholesterol transporter 1) genes as the target genes for lowering LDL-C levels, and PPARA (peroxisome proliferator-activated receptor alpha), ANGPTL3 (angiopoietin-like 3), LPL (lipoprotein lipase), and APOC3 (apolipoprotein C3) as the target genes for lowering TG levels (Table S3). To mimic the lipid-modifying effect of the drug targets, we selected SNPs within a 100 kb window region of each gene associated with relevant lipid traits at a genome-wide significance level, following the approach employed in previous studies [21, 22]. We utilized summary-level data from GWAS of HDL-C (for CETP), LDL-C (for HMGCR, PCSK9, LDLR, APOB, ABCG5/ABCG8, and NPC1L1), and TG (for LPL, PPARA, APOC3, and ANGPTL3) to identify the genetic instrument variables for these drug targets. We included SNPs that exhibited weak linkage disequilibrium (r^2 < 0.2, 250 kb). This approach was employed to maximize the precision and statistical power of the analysis. However, no significant SNP within PPARA location was found, we excluded it from further evaluation. 10 drug targets were included in final analysis: APOB, HMGCR, NPC1L1, PCSK9, ABCG5/ABCG8, LDLR, LPL, ANGPTL3, CETP, and APOC3, detail information for each target were presented in Table S3.

To examine the stability and reliability of the findings, we did additional analyses by constructing an alternative set of genetic instruments that included the role of Apo-B and Apo-A1. Apo-B, a prominent transporter implicated in the formation of LDL-C and TG, were used for constructing genetic instruments targeting HMGCR, PCSK9, NPC1L1, ABCG5/ABCG8, LDLR, LPL, ANGPTL3, APOB, and APOC3. Conversely, Apo-A1, a key transporter for HDL-C, was utilized to establish instruments for CETP and LPL.

For drug targets that exhibited suggestive significance in the MR analysis for migraine risk, publicly available expression quantitative trait loci (eQTL) data from the Genotype-Tissue Expression project (GTEx-V8) and eQTLgen were utilized. We specifically focused on tissues where the target genes were known to be highly expressed. Within these tissues, we identified cis-eQTLs that exhibited independent and statistically significant associations with the expression levels of the drug target genes. To be considered as instrumental variables (IVs) in our analysis, these cis-eQTLs had to meet stringent criteria, including a significance threshold of *P* < 5*10^–8^ and a linkage disequilibrium threshold of r^2^ < 0.1. These cis-eQTLs were considered as instrumental variables (IVs) in our analysis.

### Outcome GWAS

We collected summary-level data for migraine from two large studies. The first study sourced its data from FinnGen release 5, comprising 8,547 individuals with migraine and 176,107 controls. The mean age of the participants was 40.21 years. Migraine cases were identified using diagnostic codes from the 8th, 9th, and 10th codes of the International Classification of Diseases.

The second study, conducted by Choquet et al., involved a meta-analysis of migraine GWAS using data from the Genetic Epidemiology Research in Adult Health and Aging (GERA) cohort and UK Biobank [23]. This study included 28,852 migraine cases and 525,717 controls. Among the participants, 92.55% were of European descent, with 77.98% of cases and 53.22% of controls being female. It is worth mentioning that there was some overlap between the exposure dataset of Apo-A1 and Apo-B and the current study, accounting for up to 20.75% of the samples. However, the potential bias resulting from this overlap was deemed to be negligible, less than 1%.

In order to affirm the appropriateness of the genetic variants as drug targets, positive control analyses were carried out using coronary heart disease (CHD) as the desired outcome. The GWAS of CHD were conducted by CARDIoGRAMplusC4D.

### Statistical analysis

In the primary Mendelian randomization (MR) analysis, we utilized the inverse-variance weighted (IVW) method to estimate the causal effects of genetically proxied lipid traits and lipid-lowering targets on migraines. To facilitate interpretation and demonstrate the expected directions of effect associated with lipid-lowering medications, the odds ratios (ORs) for migraine risk were scaled to represent a 1-SD increase in HDL-C or Apo-A1, or a 1SD decrease in LDL-C, Apo-B, or TG. The gene expression data used in this study were quantified in terms of changes in expression levels per additional effect allele, which were measured as 1-standard deviation (SD). Initially, univariable MR analyses were conducted to estimate the associations between genetically predicted lipid levels and migraine risk. Following this, we conducted multivariable Mendelian randomization (MR) analysis to investigate plausible mediated pathways underlying the statistically significant associations between lipid traits and migraines. Established risk factors, namely smoking, weekly alcohol intake, body mass index (BMI), major depressive disorder, as well as systolic and diastolic blood pressure, were individually incorporated into each multivariable MR model to examine their potential mediating effects. Furthermore, we conducted fixed-effect meta-analyses to pool the IVW estimates obtained from the FinnGen consortium and the Choquet study both for each lipid trait and each target gene. To assess the relationship between the expression of drug targets and migraines, we employed the Summary data-based Mendelian randomization (SMR) method, utilizing summary data from eQTL and GWAS. To examine the influence of linkage disequilibrium on the observed association, we applied the heterogeneity in dependent instrument test. A significance level of *P* < 0.01 was used to indicate potential linkage-related associations.

To address the issue of multiple testing, we employed the Bonferroni correction to adjust the significance levels. For the testing of 5 lipid traits, we utilized a *p*-value threshold of < 0.01 (0.05 divided by 5), while for the analysis of 10 drug targets, a *p*-value threshold of < 0.005 (0.05 divided by 10) was applied. A suggestive association was considered when the uncorrected meta-analytic IVW *p*-value was < 0.05. A significant relationship with migraine risk was determined if the association showed statistical significance with a Bonferroni-corrected *p*-value < 0.05. We calculated the statistical power for the MR analysis using the mRnd website (http://cnsgenomics.com/shiny/mRnd/) (Table S10). For other analyses, we considered a two-sided *P* < 0.05 as statistically significant.

### Sensitivity analysis

We calculated the F statistics for the instruments by taking the square of the β coefficient divided by the square of the standard error. An F statistic exceeding 10 was deemed as indicative of sufficient instrument strength. To strengthen the robustness of our MR estimates, we employed four additional sensitivity analysis methods: MR Egger, weighted median method, simple mode method, and weighted mode method. The presence of horizontal pleiotropy was assessed using the Egger-intercept test. Heterogeneity among the included SNPs was evaluated through Cochrane's Q-test and the leave-one-out method. For significant MR associations with drug targets, we applied more stringent LD thresholds (r2 < 0.1, r2 < 0.01, and r2 < 0.001) to test the robustness of our findings.

To address the possibility of confounding through linkage disequilibrium, where a variant closely correlated with the true causal variant may affect the outcome through a non-lipid pathway, we conducted a Bayesian colocalization analysis. This analysis helps to examine the potential for genetic confounding by assessing the posterior probabilities of distinct causal variants, shared causal variant, and the probability of colocalization, given the presence of a causal variant for the outcome. Bayesian colocalization was used with the default prior probabilities of 10^–4^, 10^–4^ and 10^–5^ for a variant within the relevant genomic locus being associated with the exposure trait, outcome trait, or both traits, respectively. The Bayesian colocalization analysis provides several outputs of interest. These include the posterior probability of distinct causal variants (H3), which indicates the probability that there are separate causal variants for the exposure and outcome traits. The shared causal variant (H4) represents the probability that there is a single causal variant influencing both traits.

The primary output of interest is the probability of colocalization, which is the probability that the exposure and outcome traits are affected by the same causal variant, given the presence of a causal variant for the outcome. This probability is calculated as H4 divided by the sum of H3 and H4 (H4/(H3 + H4)). It provides an indication of the extent to which the same genetic variant influences both the exposure and outcome traits.

All the statistical analyses described above, including the Mendelian randomization (MR), Bayesian colocalization, and meta-analysis, were performed using R. The 'TwoSampleMR' package was utilized for MR analysis, while the 'Coloc' package was employed for Bayesian colocalization analysis. Additionally, the 'Metafor' was used for meta-analysis to combine the results from different datasets.

## Result

### Lipid trait and migraine risk

Instrumental variables for lipid traits including HDL-C, LDL-C, TG, Apo-A1, and Apo-B were presented in Tables S4–S8.

**Fig. 2:**
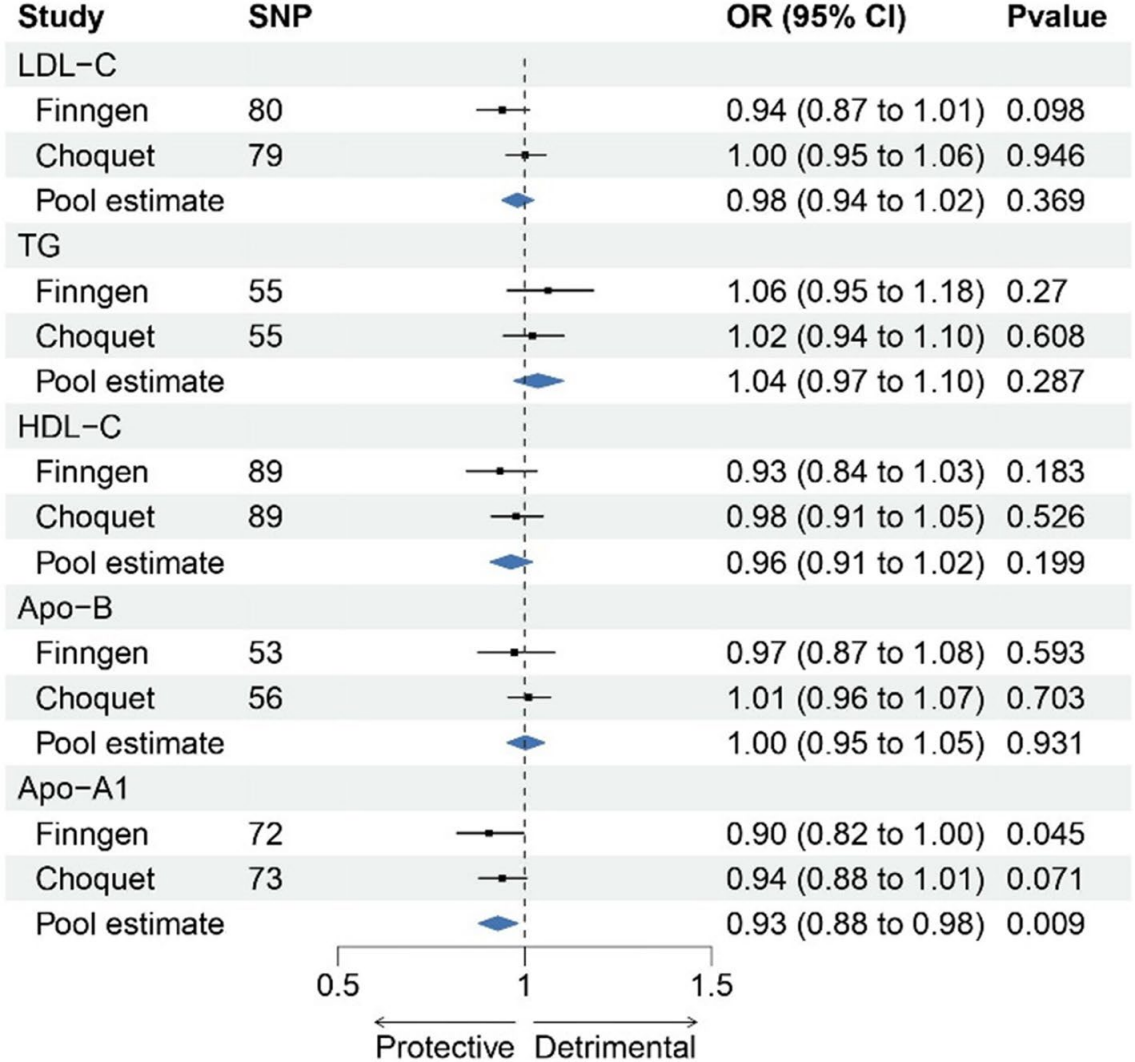
Forest plot of association of lipid traits with risk of migraine

**Fig. 3:**
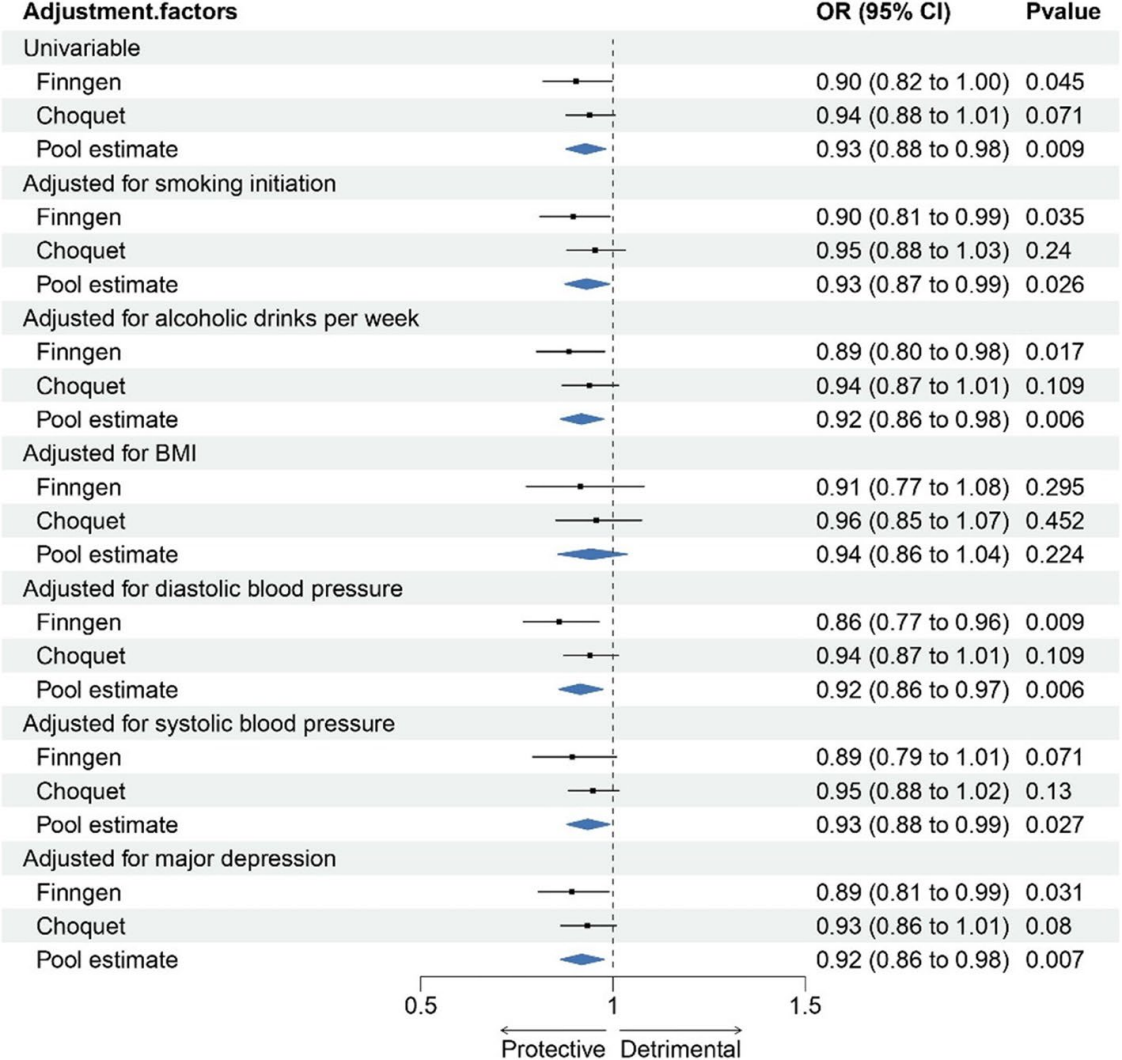
Association between genetically proxied Apo-A1 and migraine after adjustment for potential mediators

In the FinnGen GWAS, there was a suggestive association between increases in genetically proxied APO-A1 levels and a decreased risk of migraine (OR = 0.90 [95% CI, 0.82–0.99]; *p* = 0.05) (Fig. 2, Table S11). This finding was not but close to suggestive significance in the Choquet GWAS (OR = 0.94 [95% CI, 0.88–1.01]; *p* = 0.07). The effect estimates of meta-analysis reached Bonferroni-adjusted statistical significance (OR = 0.93 [95% CI, 0.88–0.98]; *p* = 0.0087). Scatter plots of association between lipid traits and migraine were presented in Figures S4-8. In the multivariate MR analysis, adjusting for BMI revealed a null association between APO-A1 and migraine, indicating a partial mediated effect of BMI in the relationship (Fig. 3). Most causal associations remained consistent even after adjusting for confounding factors such as smoking, alcoholic drinks per week, major depressive disorder, and blood pressure. However, no significant associations were found between other lipid traits and migraine risk.

The conducted sensitivity analyses provided further support for the reliability of the findings. The MR-Egger method, which assesses the presence of horizontal pleiotropy, did not indicate any signs of pleiotropic effects in the five lipid traits examined (Table S14). Nonetheless, notable heterogeneity was observed between the 5 lipid traits and the Choquet dataset, while no significant heterogeneity was found between these lipid traits and the FinnGen dataset, except for HDL and Finngen dataset (Table S14).

### Genetically predicted primary lipid-modifying effect of targets and migraine risk

Instrumental variables for lipid-modifying drug targets including HMGCR, NPC1L1, PCSK9, APOB, ABCG5 and ABCG8, LDLR, ANGPTL3, APOC3, CETP, and LPL were presented in Table S9. In our positive control analyses, we observed significant associations between genetically proxied drug targets and a reduced risk of CHD, indicating the effectiveness of the genetic instruments, except for ANGPTL3 and NPC1L1, which showed a tendency towards protection but did not reach statistical significance. These findings provide further confirmation of the effectiveness of the genetic instruments. Figure S1, which was consistent with previous studies. The F statistics of the genetic instruments utilized in the analyses exceeded the threshold of 10, signifying a satisfactory level of instrument strength (Table S10).

**Fig. 4:**
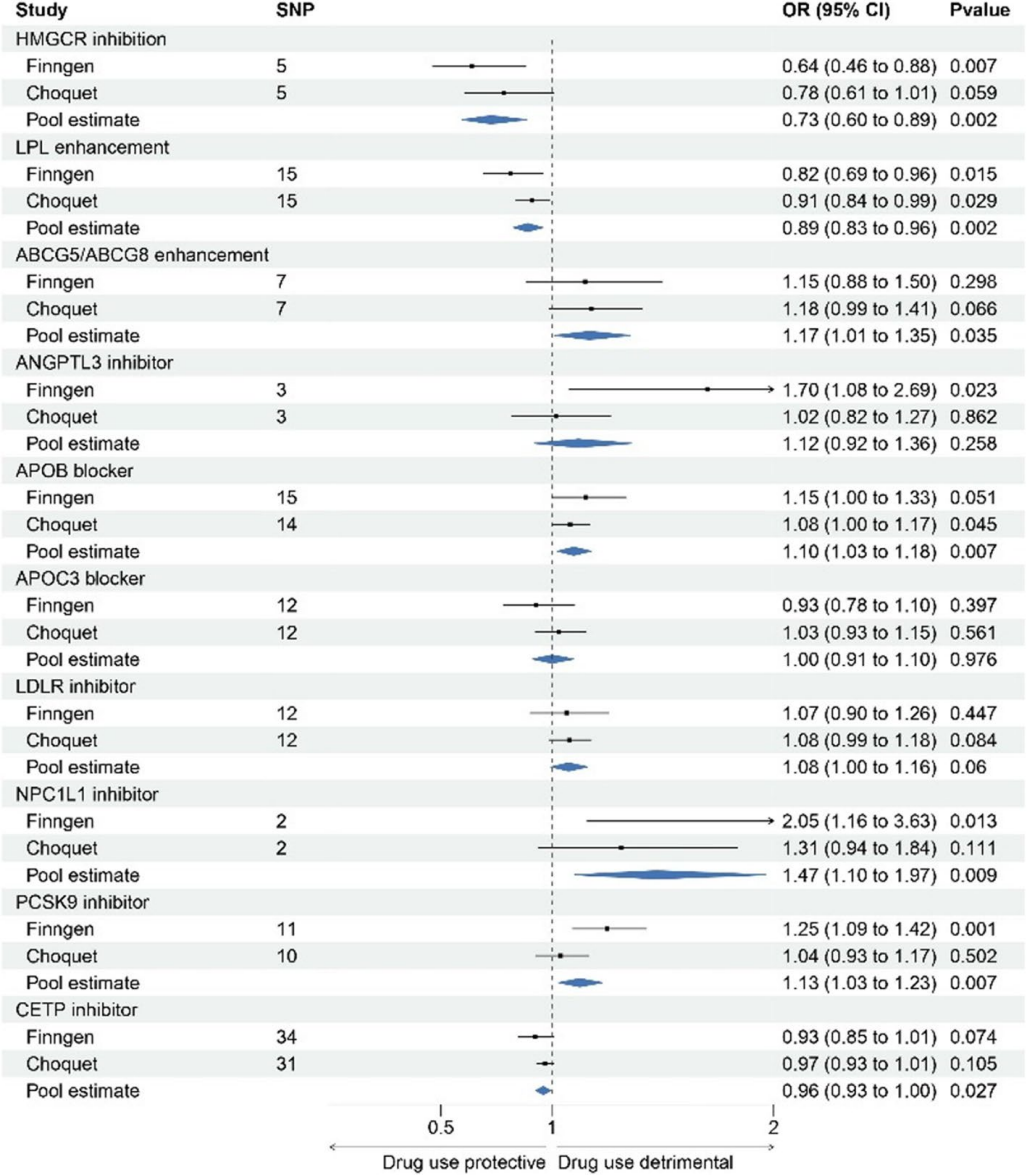
Forest plot of association of genetically proxied drug targets with risk of migraine using primary pharmacological effect

Figure 4 and Table S12 showed the associations between 10 lipid-lowering drug classes and migraine risk. Scatter plots of association between those drug targets and migraine risk were presented in Figures S9-18. HMGCR inhibition, corresponding to a 1-SD reduction in LDL-C, was significantly causally associated with a lower risk of migraine (FinnGen dataset: OR = 0.64, [95% CI, 0.46–0.88], *p* = 0.0006; Choquet dataset: OR = 0.78, [95% CI, 0.60–1.01], *p* = 0.06; pooled estimates: OR = 0.73, [95% CI, 0.60–0.89], *p* = 0.0016). Similarly, LPL enhancement, corresponding to a 1-SD reduction in TG, was significantly causally associated with a lower risk of migraine (FinnGen dataset: OR = 0.82, [95% CI, 0.69–0.96], *p* = 0.01; Choquet dataset: OR = 0.91, [95% CI, 0.83–0.99], *p* = 0.03; pooled estimates: OR = 0.89, [95% CI, 0.83–0.96], *p* = 0.002).

In contrast, ABCG5/ABCG8 enhancement, APOB inhibition, NPC1L1 inhibition, PCSK9 inhibition was found to be suggestively associated with higher risk of migraine. Conversely, CETP inhibition demonstrated a suggestive association with a lower risk of migraine.

No significant associations were observed between genetically predicted primary lipid levels modified by ANGPTL3, APOC3, and LDLR, and the risk of migraine.

**Fig. 5:**
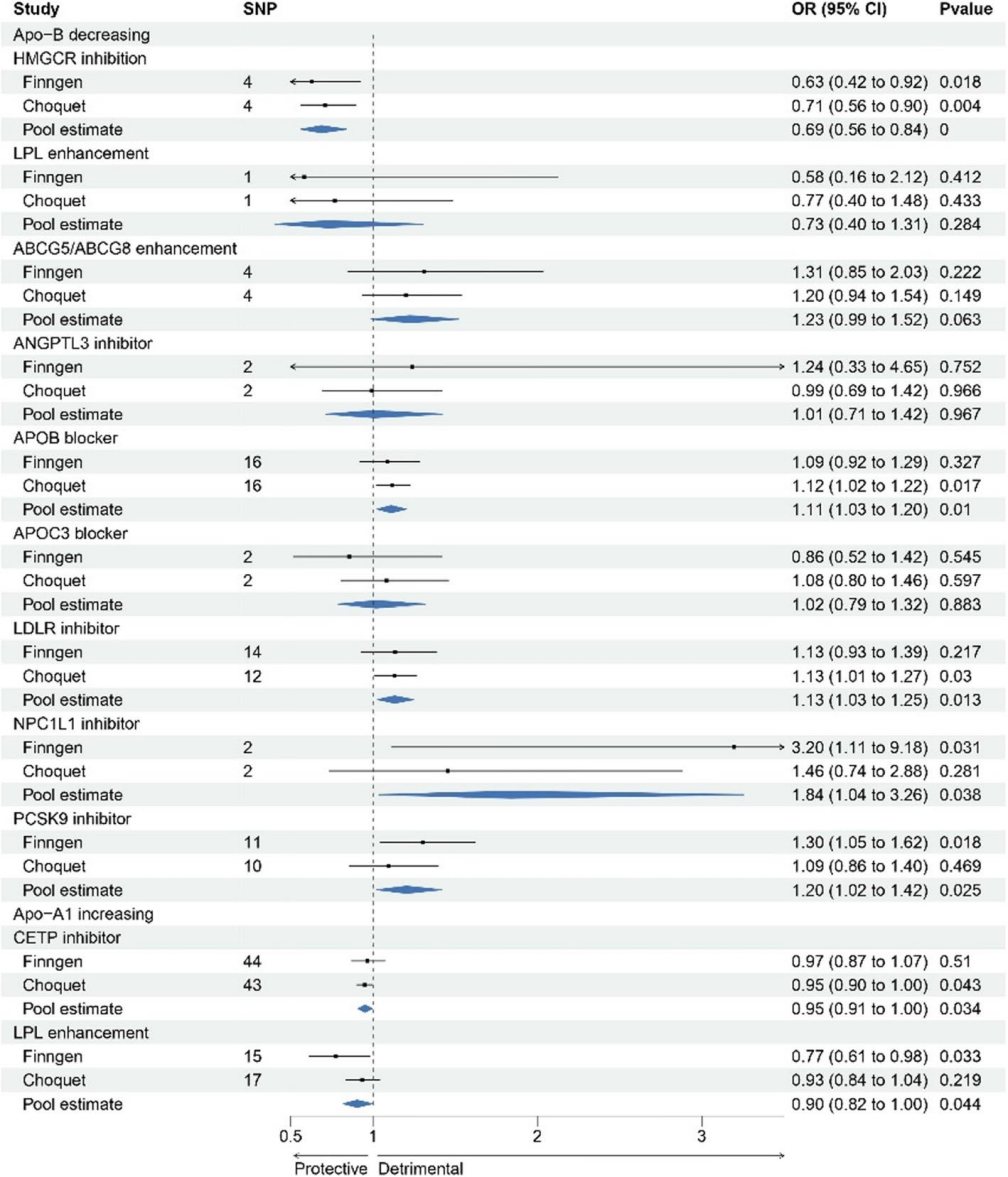
Forest plot of association of genetically proxied drug targets with risk of migraine using alternative pharmacological effect

Genetic mimicry of target gene effect weighted on Apo-B or Apo-A1 also retrieved similar results (Fig. 5, Table S13), although genetic mimicry of LDLR inhibition equivalent to 1 SD Apo-B decrease was suggestively associated with higher migraine risk (Finngen dataset: OR = 1.13, [95% CI, 0.93–1.39], *p* = 0.22; Choquet dataset: OR = 1.13, [95% CI, 1.01–1.27], *p* = 0.03; pooled estimates: OR = 1.13, [95% CI, 1.03–1.25], *p* = 0.01). The causal association between genetic mimicry of LPL enhancement using Apo-B level and migraine was not statistical significance because of the limited number of available instruments for this analysis.

The findings from the other MR methods showed consistent results, as presented in Table S12. The MR-Egger intercept test did not find any evidence of pleiotropy, which strengthens the validity of causal inferences (Table S15). Furthermore, the robustness of the findings was confirmed by the leave-one-out sensitivity analysis, as depicted in Figures S2-3. Additional analyses were conducted using much lower LD thresholds (r^2^ < 0.1, r^2^ < 0.01 and r^2^ < 0.001) for HMGCR and LPL. These analyses yielded results that were in agreement with our primary findings, albeit with reduced statistical power due to the exclusion of multiple SNPs (Tables S16-S17).

### Target gene expression and migraine risk

The results from the SMR analysis investigating the association between the expression of HMGCR, CETP, LPL, APOB, NPC1L1, ABCG5/ABCG8, and PCSK9 and migraine provided additional evidence, but most of the associations did not reach statistical significance. It is important to note that the relatively small sample size of the eQTLs data may have resulted in insufficient statistical power for detecting certain associations. Consistent with the findings from the drug target MR analyses mentioned earlier, the SMR analyses showed that higher expression level of PCSK9, APOB, and LPL was associated with a lower risk of migraine, while higher expression of HMGCR, ABCG5/ABCG8, and CETP could contribute to a higher risk of migraine. These results suggest that the inhibition of HMGCR and CETP and the activation of LPL may have a protective effect against migraine, while the inhibition of PCSK9, APOB, and NPC1L1 and the activation of ABCG5/ABCG8 may contribute to migraine (Table S18).

### Colocalization

For LDL-C and migraine, the probability of distinct variants (0.09%) within the HMGCR gene was much less than the posterior probability of a shared causal variant (3.91%). The probability of colocalization, conditional on the presence of a causal variant for the outcome, was 97.67%. For Apo-B and migraine within the HMGCR gene, the respective probabilities were 9.90%, 0.21%, and 97.92%. For TG and migraine within the LPL gene, the respective posterior probabilities were 1.45%, 0.35%, and 80.73%. For Apo-B and migraine within the LPL gene, the respective posterior probabilities were 1.36%, 0.59%, and 69.64%. These findings provide evidence that the effects of HMGCR and LPL on migraine are not possible to be influenced by confounding from a variant in LD. Other colocalization results between gene targets and migraine are presented in Table S19.

## Discussion

This research study provided compelling genetic evidence supporting the potential of HMGCR inhibition and LPL enhancement in reducing the risk of migraines. These findings suggested that HMGCR and LPL could serve as promising drug targets for migraine prevention. Importantly, the observed risk reduction appeared to be independent of LDL-C or TG control, as there was no clear evidence of a general effect of LDL-C or TG on migraine risk. The robustness of the results was confirmed through various approaches of constructing genetic instruments and the use of two independent migraine datasets. Furthermore, the study identified weak evidence suggesting that CETP inhibition may offer protection against migraines. Additionally, the activation of ABCG5/ABCG8 and the inhibition of APOB, NPC1L1, PCSK9, and LDLR were found to suggestively contribute to migraine. These findings shed light on potential molecular mechanisms underlying migraine and provide insights into additional therapeutic targets that could be explored for migraine management.

The available evidence did not support TG, LDL-C, HDL-C, and Apo-B as causal risk factors for migraine, which was consistent with previous studies [11, 12, 24, 25]. In a study conducted by Siewert et al., a significant genetic correlation between lipid traits and migraine was reported. However, none of the Mendelian randomization experiments investigating the causal relationship between lipid phenotypes and migraine reached statistical significance in their study [12]. Guo et al. conducted a Mendelian randomization analysis and found no significant causal association between high-density and low-density lipoprotein particles and migraine. However, they did observe nominal instrumental effects on migraine for triglyceride-rich lipoprotein particles, which did not remain did not remain significant after adjusting for multiple testing [11].

Our study yielded noteworthy findings regarding the causal associations between Apo-A1 levels and migraine. We observed significant associations, however, after adjusting for BMI, the strength of these associations attenuated, indicating a potential mediation effect of BMI on the relationship between Apo-A1 and migraine. Intriguingly, when we adjusted for other factors such as smoking initiation, alcoholic drinks, major depression, and blood pressure, the associations between Apo-A1 and migraine remained relatively unchanged. This suggests that these factors may not significantly mediate the relationship between Apo-A1 and migraine. Nevertheless, further investigations are warranted to comprehensively understand the underlying mechanisms and potential mediators involved in the association between Apo-A1 levels and migraine.

Statins, a class of lipid-lowering drugs, are widely prescribed worldwide for the prevention and treatment of CHD and ischemic stroke. These medications exert their therapeutic effects by inhibiting the activity of HMG-CoA reductase, which is encoded by the HMGCR gene. Through this mechanism, statins effectively reduce the production of cholesterol in the liver, leading to decreased levels of LDL cholesterol in the bloodstream [14, 26]. Due to their established efficacy and safety profile, statins have become a cornerstone in the management of CHD [26]. The clinical relevance of HMGCR in relation to migraines has been supported by multiple studies, including randomized controlled trials, epidemiological investigations, laboratory experiments, and genetic analyses. A previous study identified a consistent genetic signal between various subfractions of lipoproteins and migraines. This signal was located on the chromosomal region 5q13.3 and was found to colocalize with the HMGCR gene in both circulatory and musculoskeletal tissue [11]. Buettner and Burstein conducted a cross-sectional study involving 5,938 individuals, where they observed an interesting association between serum vitamin D levels, statin use, and the occurrence of severe headaches or migraines. Specifically, they found that individuals with higher serum vitamin D levels who also used statins had significantly reduced odds of experiencing severe headaches or migraines [17]. Furthermore, Additionally, several randomized controlled trials have provided evidence for the efficacy of statins in the prevention of migraines. Buettner et al. suggested that simvastatin plus vitamin D could effectively prevent migraines in adults with episodic migraines while also being well-tolerated [16]. Hesami et al. and Marfil-Rivera et al. used atorvastatin and found positive outcomes [27, 28]. Additionally, Sahebnasagh et al. found that combining sodium valproate with atorvastatin resulted in a reduction in the number of attacks and pain severity [29]. Mazdeh et al. reported a significant reduction in the number of attacks when using propranolol and rosuvastatin [30]. In addition to the potential in alleviating the attack frequency and severity of migraines, the use of statins in migraine prevention may have the added advantage of addressing endothelial dysfunction, a condition associated with CHD and ischemic stroke. These findings suggest that statins not only have a direct impact on migraine symptoms but also offer potential cardiovascular benefits, making them a promising therapeutic option for individuals with migraines who are at risk for cardiovascular complications. More high-quality randomized, placebo-controlled trials are warranted to provide more robust and conclusive findings. Besides, our findings suggested that the mechanisms extend beyond lowering LDL cholesterol levels. HMGCR inhibitors' effects on immunomodulation, neuroprotection, and vascular function may play pivotal roles. Immunomodulatory effects of HMGCR inhibitors may suppress the production of inflammatory mediators and cytokines, and mitigate inflammatory responses [31–33]. These effects may be particularly relevant, as it has been found that patients with chronic migraines often have elevated levels of certain inflammatory markers, such as C-reactive protein (CRP), interleukin-6 (IL-6), and tumor necrosis factor-alpha (TNF-α), which may suggest an underlying systemic inflammatory state, and activation of the trigeminal vascular system leads to a cascade of inflammatory responses, causing neurogenic inflammation, resulting in the sensitization of peripheral and central pain pathways, potentially triggering a migraine attack [34–37]. Moreover, HMGCR inhibitors' neuroprotective effects, including the reduction of oxidative stress, might contribute to migraine prevention [38–40]. Additionally, improvements in vascular function, such as enhanced endothelial function, induced by statins, could be beneficial in mitigating migraines [41–44]. While these mechanisms provide potential pathways through which HMGCR inhibitors might reduce migraine risk, the exact roles and interactions of these mechanisms still require further exploration and validation.

This study presents novel findings regarding the causal association between the enhancement of LPL activity and the risk of migraines. To the best of our knowledge, this study is the first to establish such a relationship. Enhanced LPL activity could lead to increased level of Apo-A1 in a previous Mendelian randomization study [45]. Interestingly, our study revealed a causal association between lower Apo-A1 levels and a reduced risk of migraine, the increased LPL activity could contribute to alterations in Apo-A1 levels, which in turn may influence the risk of migraine. LPL is expressed in various tissues, including the central nervous system and blood vessels. It was found that microglia lacking LPL exhibited excessive accumulation of lipid droplets and a pro-inflammatory lipid profile [46, 47]. LPL activity has been associated with the regulation of inflammatory pathways [48]. Migraine is recognized as a neuroinflammatory disorder, and inflammation contributes to the activation of trigeminovascular pathways implicated in migraine pathophysiology [49–51]. Enhanced LPL activity may suppress the inflammatory response, attenuating neuroinflammation and dampening the inflammatory cascade associated with migraines. Additional studies involving in vitro experiments, animal models, and clinical trials are needed to unravel the intricate molecular pathways and confirm the therapeutic potential of LPL modulation in reducing migraine occurrence.

Our findings suggested the possibility, without confirmation, that exposure to bile acid sequestrants targeting ABCG5/ABCG8, cholesterol absorption inhibitors targeting NPC1L1, and Mipomersen targeting APOB might increase the likelihood of migraines in individuals. However, the observed relative risk magnitude is smaller than the estimated degree of protection against CAD. Since these medications have obtained regulatory approval in multiple countries, it is feasible to conduct pharmacoepidemiologic studies using large samples from national or insurance-based prescription registers. Such studies could compare the consequences of using these medications with other lipid-lowering agents in terms of migraine risk. Additionally, it would be valuable to follow up on the differences in migraine risk among individuals who participated in previous trials.

When interpreting the results of this study, several limitations should be taken into consideration. Firstly, it should be noted that the genetic variants used in this study reflect the long-term effects of lipid level changes on migraine risk and may not directly translate to the immediate effects of lipid-lowering medications. Mendelian randomization analysis provides insights into the direction of associations rather than precise quantitative estimates. Nonetheless, these findings provide valuable insights for migraine prevention, and further clinical investigations are needed to validate these observations. Secondly, this study specifically focuses on the intended effects of drug targets and does not estimate potential unintended off-target effects. It is important to acknowledge that medications can have multiple effects beyond their intended targets, and these should be considered when interpreting the findings. Thirdly, despite conducting various sensitivity analyses that demonstrated the robustness of the findings, it is important to note that the presence of horizontal pleiotropy, where genetic variants affect both the exposure and outcome through different pathways, cannot be completely ruled out. This could potentially introduce bias into the results. Fourthly, the SMR analyses conducted in this study, which combined drug target eQTL and migraine GWAS data, yielded imprecise results across different tissues. For instance, the study observed a significant association between a genetically predicted lower expression of HMGCR in muscle skeletal tissue, but not in blood, and a decreased risk of migraines. Likewise, there was no significant association observed between genetically predicted LPL expression in either blood or adipose tissue and the risk of migraines. However, it is important to note that these associations generally exhibited similar trends, despite some not reaching statistical significance. This indicates that the limited statistical power resulting from the small sample size of eQTL data may have influenced these findings. Fifthly, the colocalization analyses conducted in this study showed relatively low posterior probabilities of shared causal variants. These findings may be attributed to factors such as limited statistical power or the absence of the causal variant in both the exposure and outcome GWAS datasets. These findings may be influenced by factors such as the sample size and statistical power. Fifthly, in our Mendelian Randomization study, we have employed SNPs as instruments with varied statistical power and effect sizes on the lipid traits. The statistical power across our drug targets and odds ratios displays a certain degree of heterogeneity, as indicated in our results (Table S10). Drug targets like PCSK9, APOB, and LPL show high statistical power across all considered odds ratios, providing robustness to our results. However, targets such as NPC1L1 exhibit lower statistical power, particularly at smaller odds ratios, emphasizing the necessity for a cautious interpretation of associated results. While all chosen genetic variants appear to be strong instruments, for those explaining a lower percentage of variance in lipid traits, there may be increased uncertainty in the results. Further studies with larger sample size and higher statistical power are needed to confirm or disprove the findings of those drug targets with low certainty. Lastly, it is crucial to acknowledge that the findings of this study pertain to individuals of European ancestry. Therefore, caution should be exercised when extrapolating these results to other ethnic groups, as genetic associations and environmental factors may vary among populations. To establish the generalizability of these findings, further research involving diverse populations is warranted.

In summary, this study did not find evidence supporting lipid traits such as TG, LDL-C, HDL, and Apo-B as causal risk factors for migraines. However, a potential causal association was observed between higher levels of Apo-A1 and a reduced risk of migraines. Additionally, this study highlights HMGCR and LPL as promising drug targets for the treatment of migraines. Further research is needed to better understand the underlying mechanisms, and the potential effectiveness of HMGCR inhibitors and LPL activators in migraine treatment should be evaluated through preclinical and clinical trials.

### Supplementary Information

**Additional file 1: Figure S1.** Forest plot of association of genetically proxied drug targets with risk of coronary heart disease using primary pharmacological effect.

**Additional file 2: Figure S2.** Plots of “leave-one-out” analyses for MR analyses of the causal effect of HMGCR on migraine in (a) Finngen dataset (b) Choquet dataset.

**Additional file 3: Figure S3.**Plots of “leave-one-out” analyses for MR analyses of the causal effect of LPL on migraine in (a) Finngen dataset (b) Choquet dataset.

**Additional file 4: Figure S4.** Scatter plot of the association between LDL and migraine in (a) Finngen dataset (b) Choquet dataset.

**Additional file 5: Figure S5.** Scatter plot of the association between HDL and migraine in (a) Finngen dataset (b) Choquet dataset.

**Additional file 6: Figure S6.** Scatter plot of the association between TG and migraine in (a) Finngen dataset (b) Choquet dataset.

**Additional file 7: Figure S7.** Scatter plot of the association between Apo-A1 and migraine in (a) Finngen dataset (b) Choquet dataset.

**Additional file 8: Figure S8.** Scatter plot of the association between Apo-B and migraine in (a) Finngen dataset (b) Choquet dataset.

**Additional file 9: Figure S9.** Scatter plot of the association between LDL and migraine using SNPs within or near the HMGCR locus in (a) Finngen dataset (b) Choquet dataset.

**Additional file 10: Figure S10.** Scatter plot of the association between LDL and migraine using SNPs within or near the NPC1L1 locus in (a) Finngen dataset (b) Choquet dataset.

**Additional file 11: Figure S11.** Scatter plot of the association between LDL and migraine using SNPs within or near the ABCG5/ABCG8 locus in (a) Finngen dataset (b) Choquet dataset.

**Additional file 12: Figure S12.** Scatter plot of the association between LDL and migraine using SNPs within or near the APOB locus in (a) Finngen dataset (b) Choquet dataset.

**Additional file 13: Figure S13.** Scatter plot of the association between LDL and migraine using SNPs within or near the PCSK9 locus in (a) Finngen dataset (b) Choquet dataset.

**Additional file 14: Figure S14.** Scatter plot of the association between LDL and migraine using SNPs within or near the LDLR locus in (a) Finngen dataset (b) Choquet dataset.

**Additional file 15: Figure S15.** Scatter plot of the association between TG and migraine using SNPs within or near the LPL locus in (a) Finngen dataset (b) Choquet dataset.

**Additional file 16: Figure S16.** Scatter plot of the association between TG and migraine using SNPs within or near the APOC3 locus in (a) Finngen dataset (b) Choquet dataset.

**Additional file 17: Figure S17.** Scatter plot of the association between TG and migraine using SNPs within or near the ANGPTL3 locus in (a) Finngen dataset (b) Choquet dataset.

**Additional file 18: Figure S18.** Scatter plot of the association between HDL and migraine using SNPs within or near the CETP locus in (a) Finngen dataset (b) Choquet dataset.

**Additional file 19: Supplementary tables.**

## Data Availability

The original contributions presented in the study are included in the article, further inquiries can be directed to the corresponding authors.

## Abbreviation

CETP: Cholesteryl ester transfer protein
LDLR: Low density lipoprotein receptor
HMGCR: 3-Hydroxy-3-methylglutaryl-CoA reductase
ABCG5: ATP binding cassette subfamily G member 5
ABCG8: ATP binding cassette subfamily G member 8
PCSK9: Proprotein convertase subtilisin/kexin type 9
APOB: Apolipoprotein B
NPC1L1: Niemann-Pick C1 like intracellular cholesterol transporter 1
LPL: Lipoprotein lipase
PPARA: Peroxisome proliferator-activated receptor alpha
ANGPTL3: Angiopoietin-like 3
APOC3: Apolipoprotein C3
MR: Mendelian randomization
LDL-C: Low-density lipoprotein cholesterol
HDL-C: High-density lipoprotein cholesterol
Apo-A1: Apolipoprotein A1
Apo-B: Apolipoprotein B
CHD: Coronary heart disease
GWAS: Genome-wide association studies
BMI: Body mass index
IVW: Inverse-variance weighted
SMR: Summary data-based Mendelian randomization
