# Supplementary figures and images for: Lipids, lipid-modifying drug target genes and migraine: a Mendelian randomization study
Source: J Headache Pain. 2023 Aug 18;24(1):112. doi: 10.1186/s10194-023-01633-x (PMC10439594; doi:10.1186/s10194-023-01633-x)

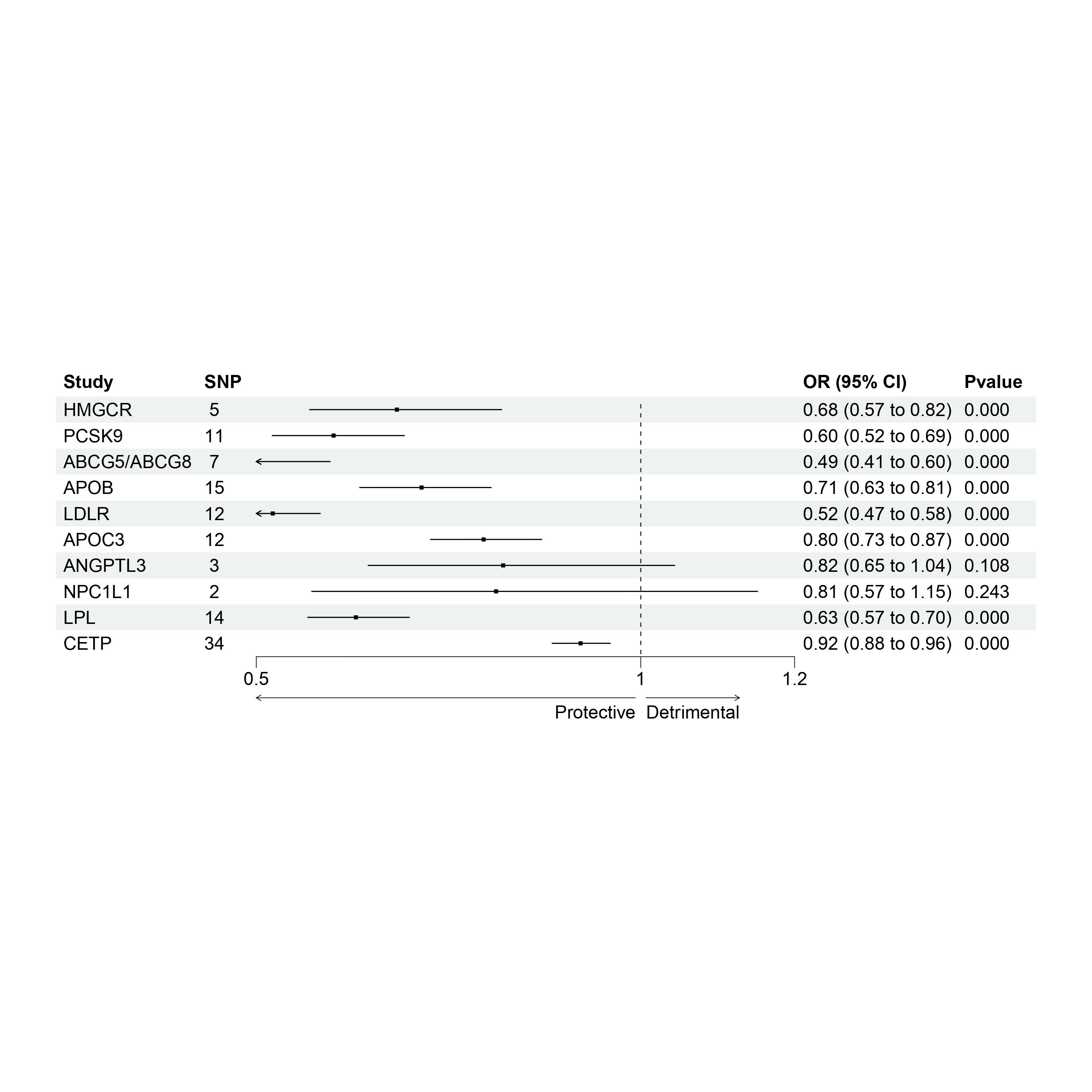

Supplement: Supplementary file 1 — Additional file 1: Figure S1. Forest plot of association of genetically proxied drug targets with risk of coronary heart disease using primary pharmacological effect. [file 10194_2023_1633_MOESM1_ESM.jpg]

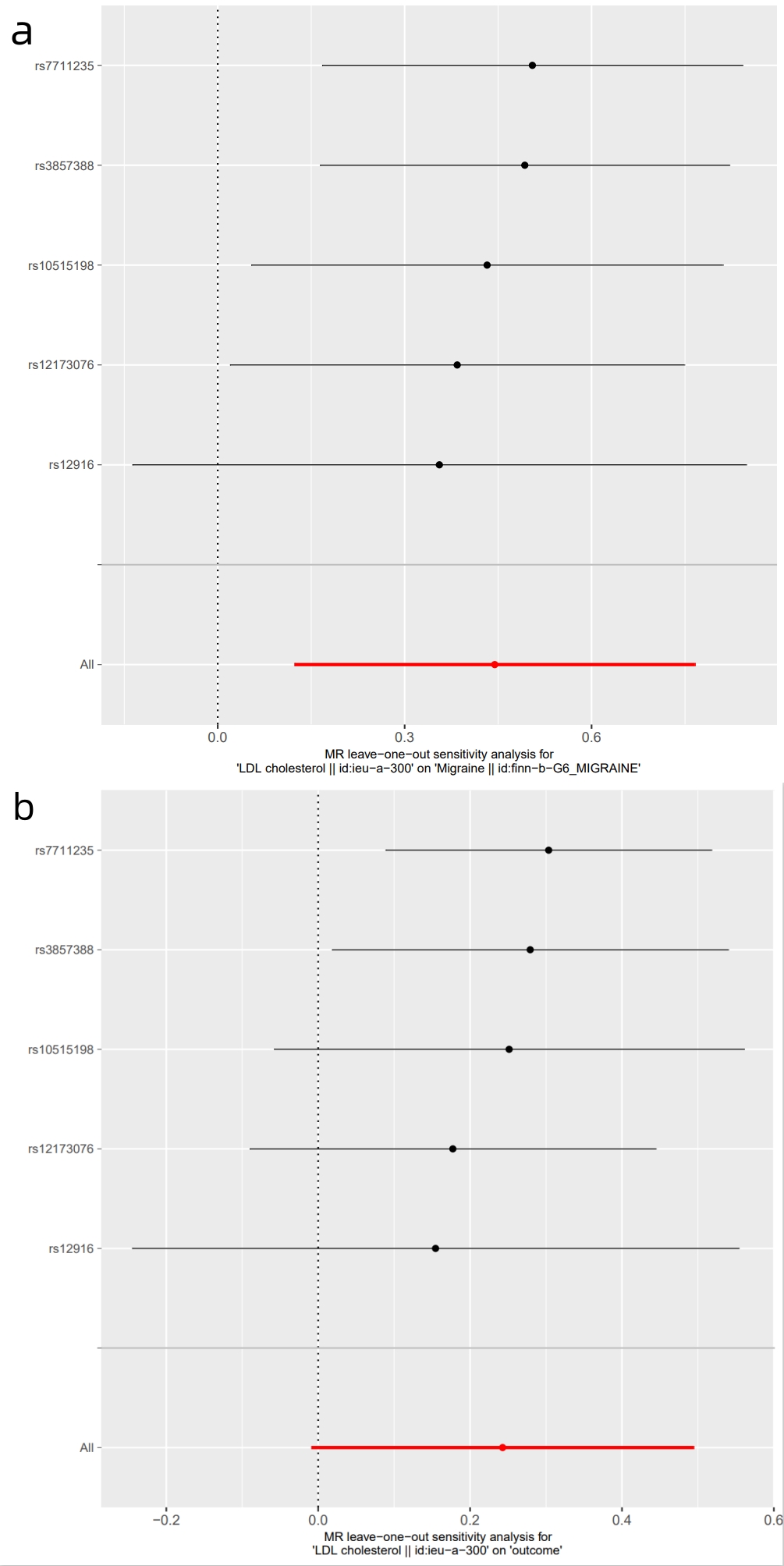

Supplement: Supplementary file 2 — Additional file 2: Figure S2. Plots of “leave-one-out” analyses for MR analyses of the causal effect of HMGCR on migraine in (a) Finngen dataset (b) Choquet dataset. [file 10194_2023_1633_MOESM2_ESM.jpg]

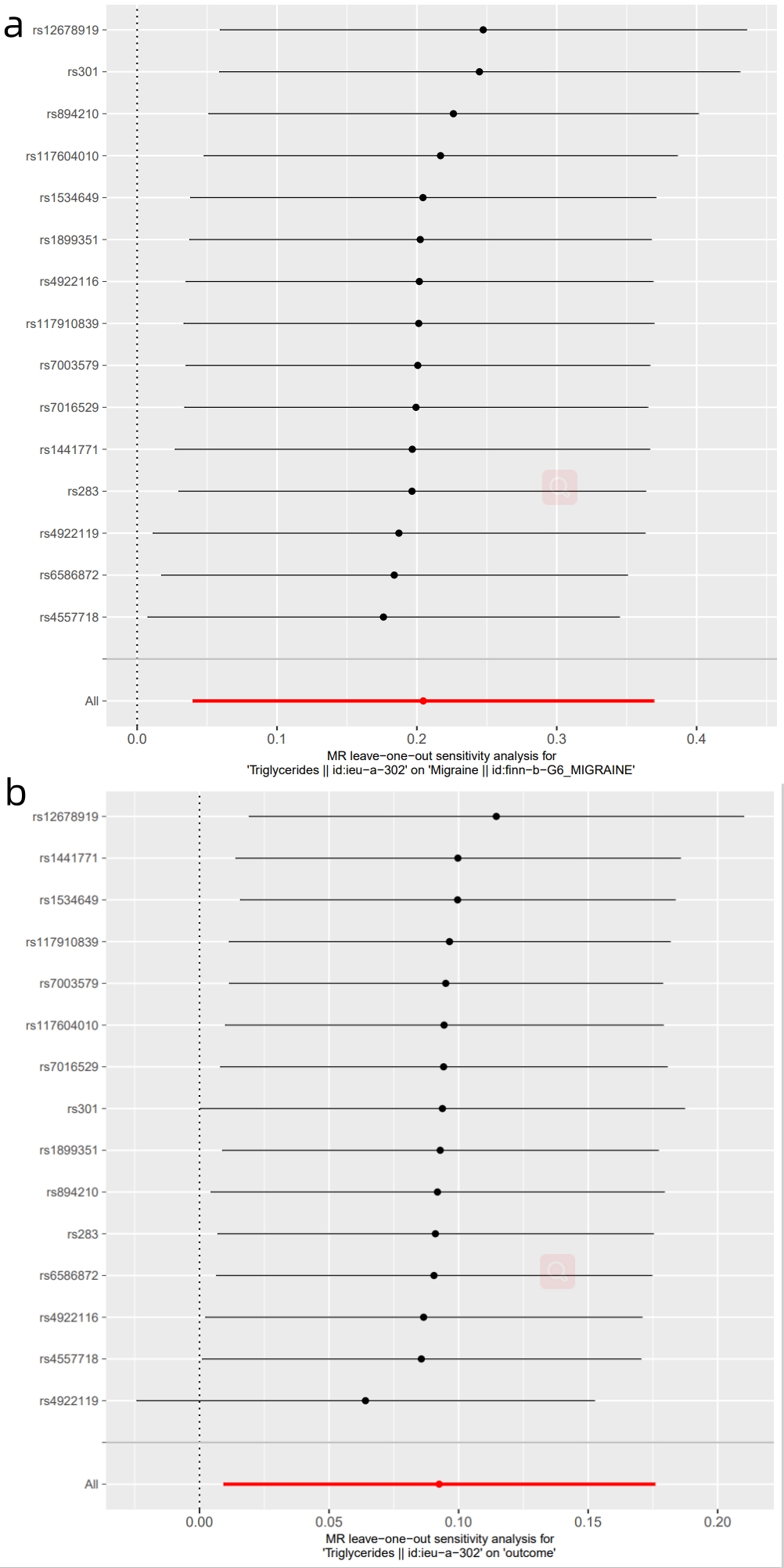

Supplement: Supplementary file 3 — Additional file 3: Figure S3.Plots of “leave-one-out” analyses for MR analyses of the causal effect of LPL on migraine in (a) Finngen dataset (b) Choquet dataset. [file 10194_2023_1633_MOESM3_ESM.jpg]

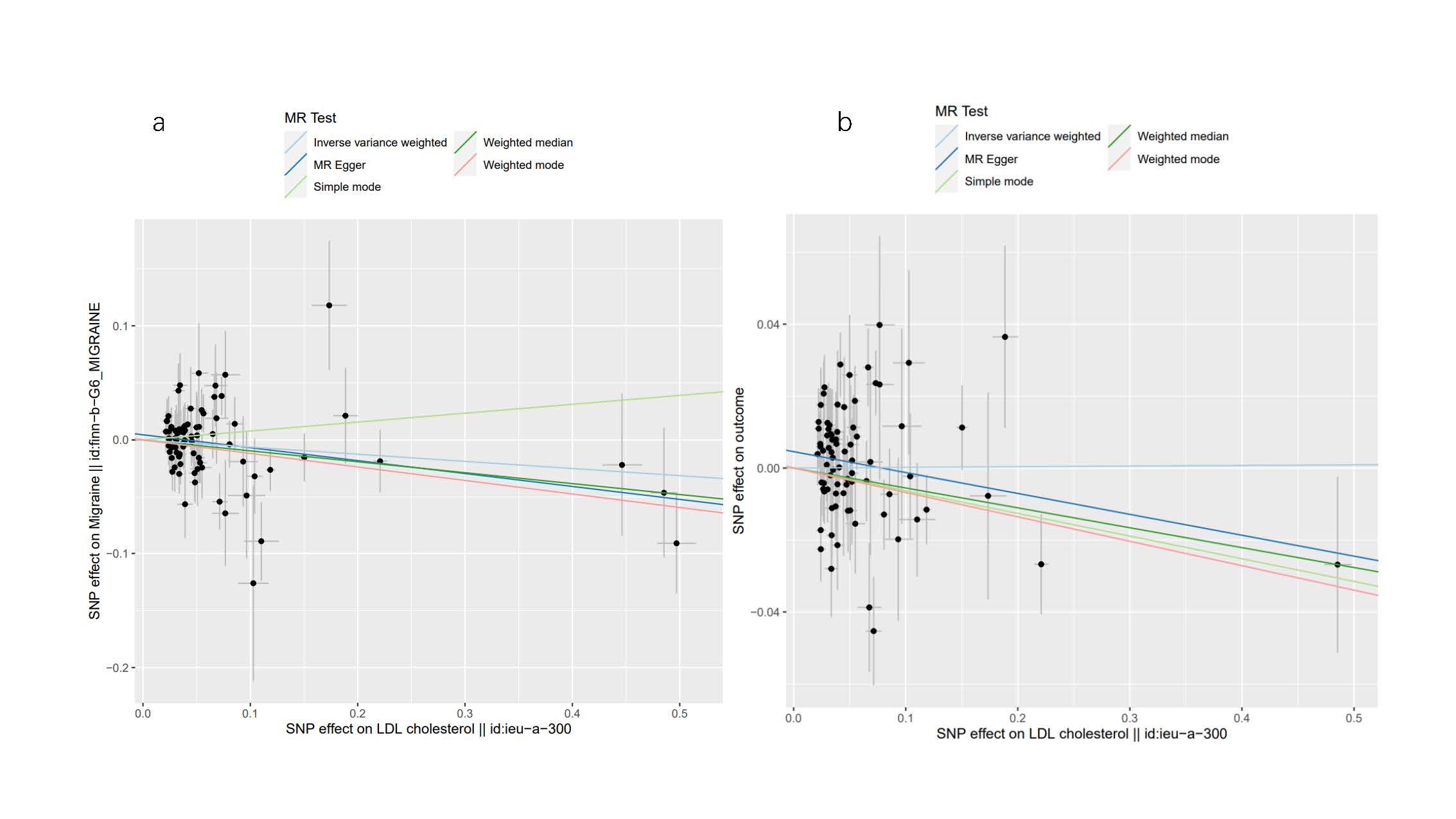

Supplement: Supplementary file 4 — Additional file 4: Figure S4. Scatter plot of the association between LDL and migraine in (a) Finngen dataset (b) Choquet dataset. [file 10194_2023_1633_MOESM4_ESM.jpg]

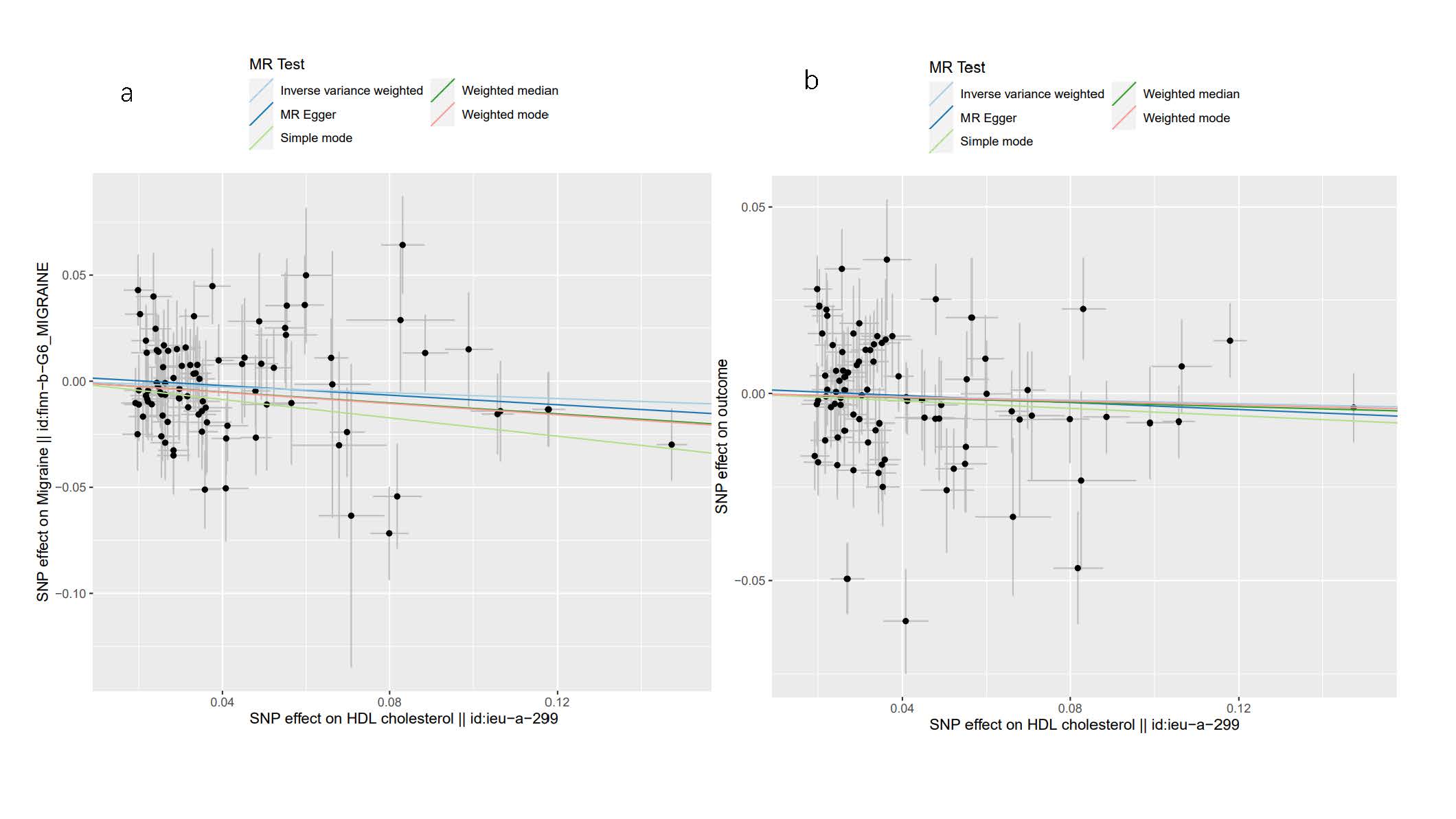

Supplement: Supplementary file 5 — Additional file 5: Figure S5. Scatter plot of the association between HDL and migraine in (a) Finngen dataset (b) Choquet dataset. [file 10194_2023_1633_MOESM5_ESM.jpg]

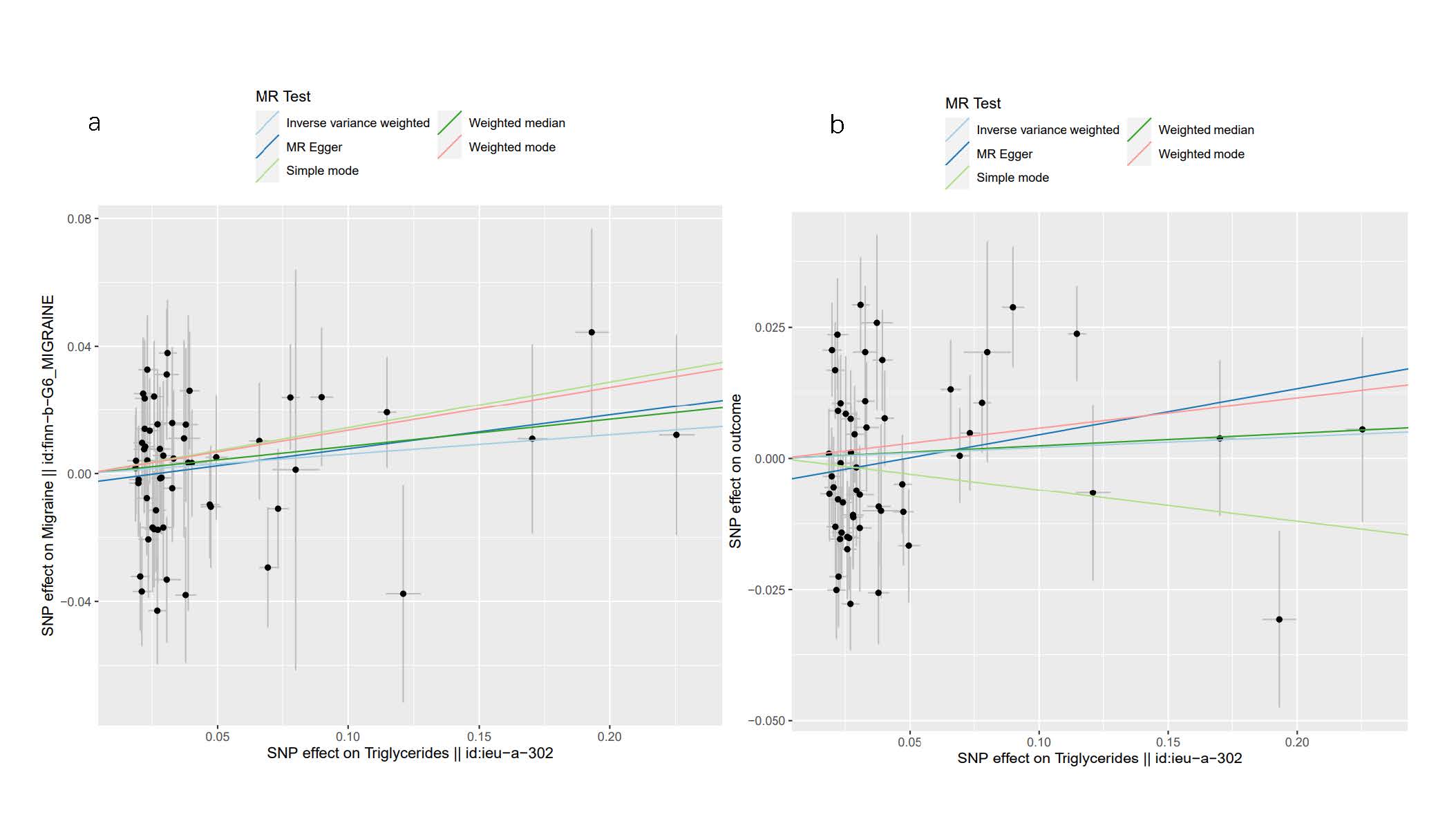

Supplement: Supplementary file 6 — Additional file 6: Figure S6. Scatter plot of the association between TG and migraine in (a) Finngen dataset (b) Choquet dataset. [file 10194_2023_1633_MOESM6_ESM.jpg]

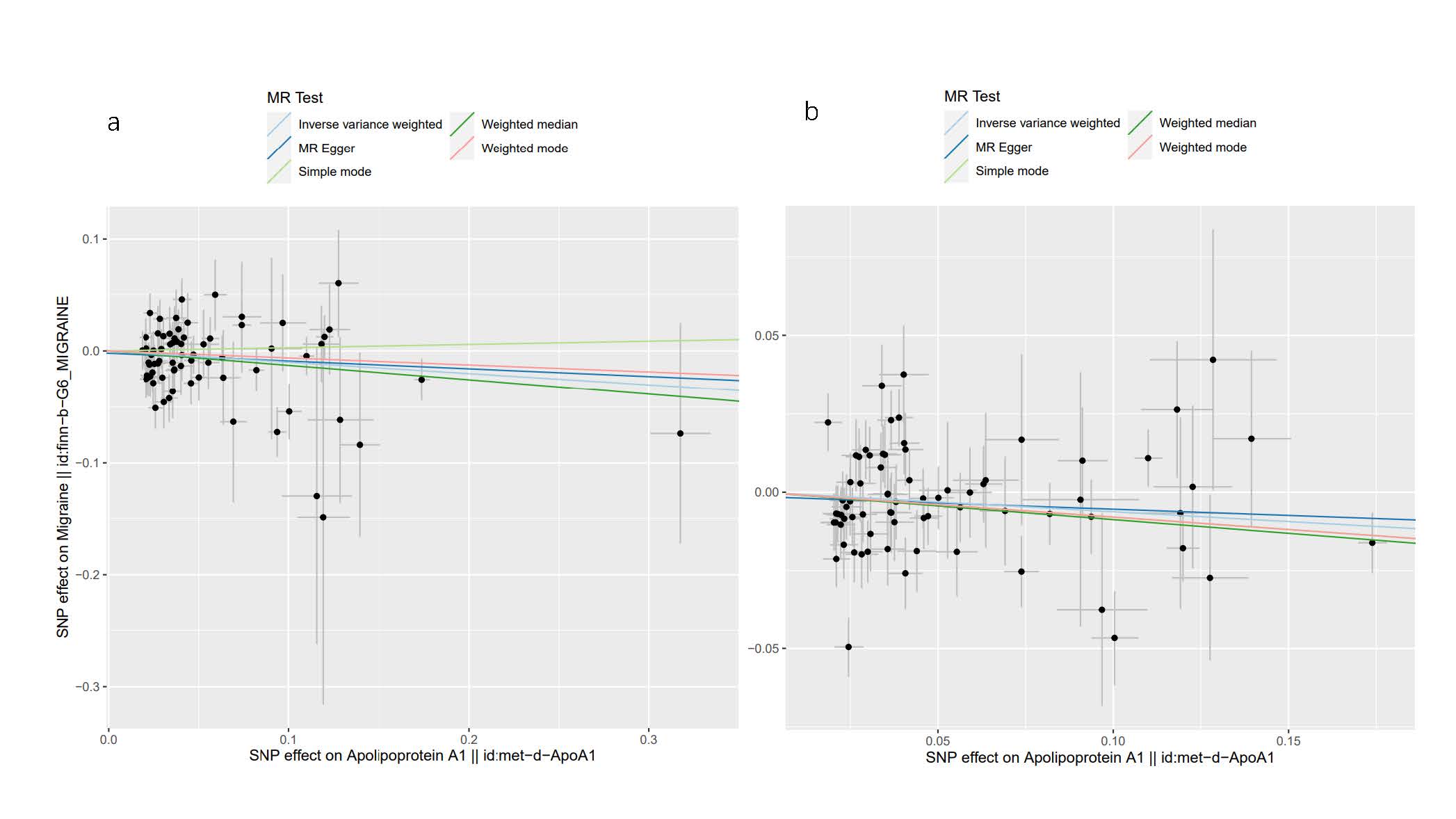

Supplement: Supplementary file 7 — Additional file 7: Figure S7. Scatter plot of the association between Apo-A1 and migraine in (a) Finngen dataset (b) Choquet dataset. [file 10194_2023_1633_MOESM7_ESM.jpg]

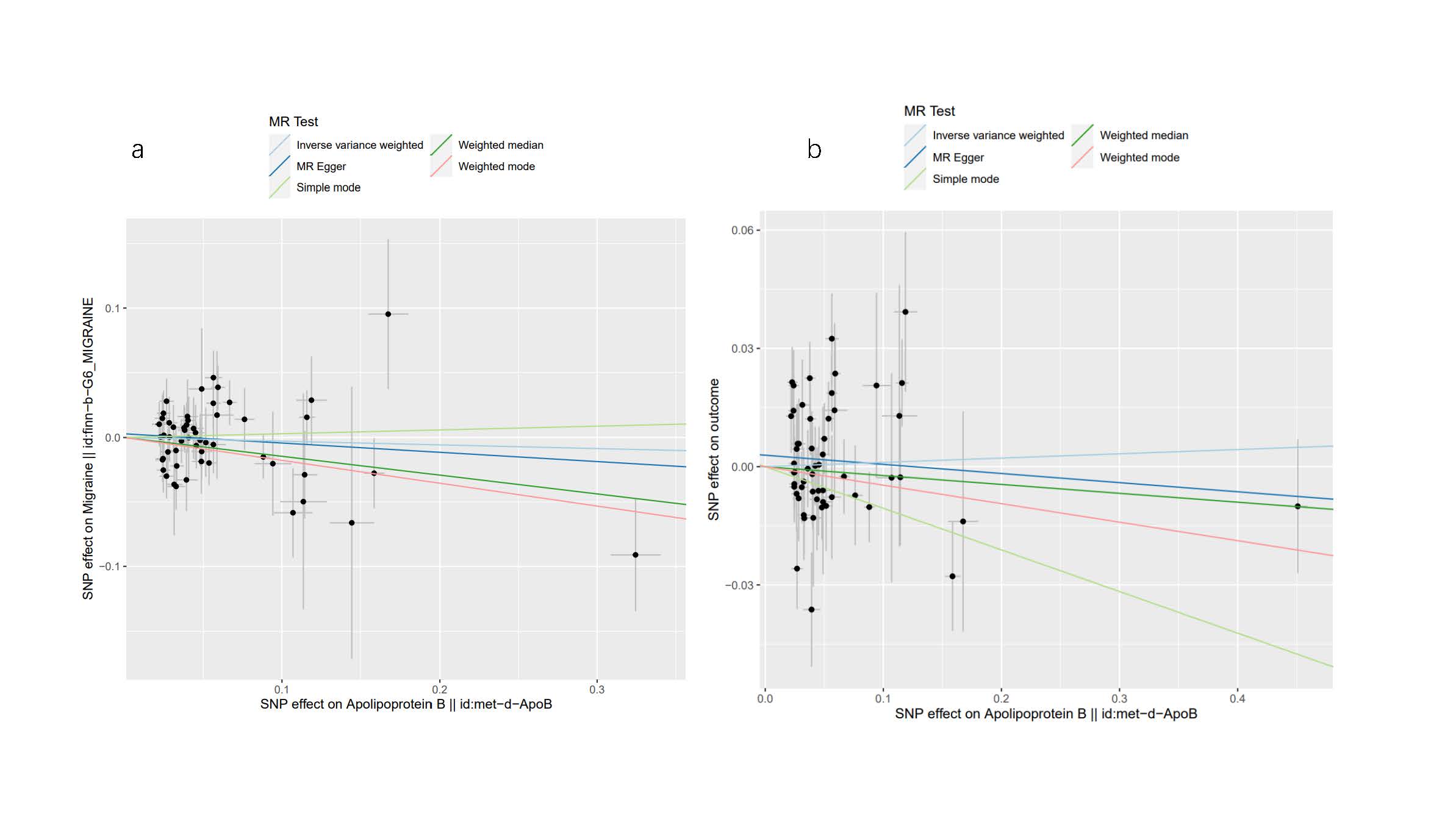

Supplement: Supplementary file 8 — Additional file 8: Figure S8. Scatter plot of the association between Apo-B and migraine in (a) Finngen dataset (b) Choquet dataset. [file 10194_2023_1633_MOESM8_ESM.jpg]

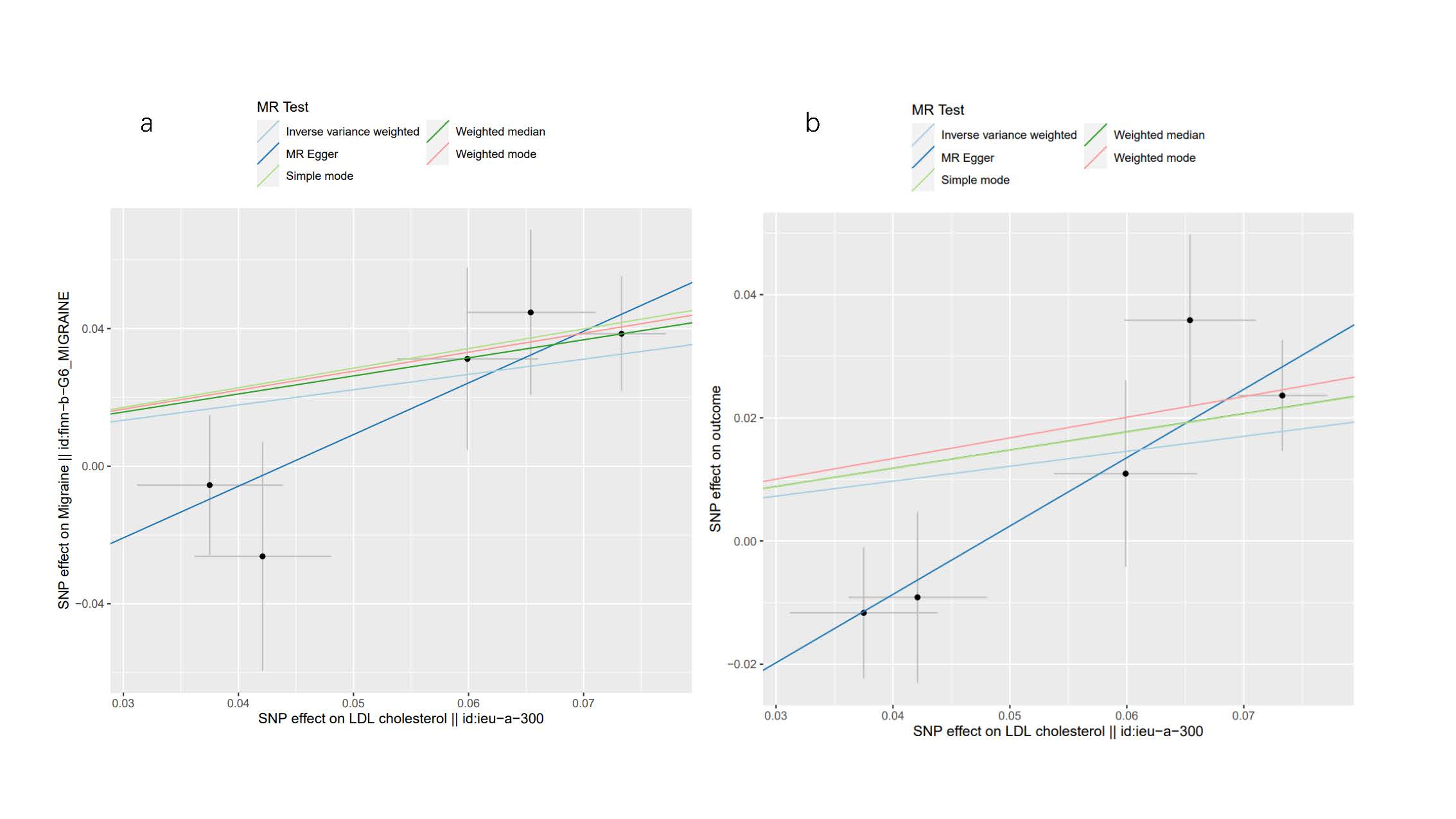

Supplement: Supplementary file 9 — Additional file 9: Figure S9. Scatter plot of the association between LDL and migraine using SNPs within or near the HMGCR locus in (a) Finngen dataset (b) Choquet dataset. [file 10194_2023_1633_MOESM9_ESM.jpg]

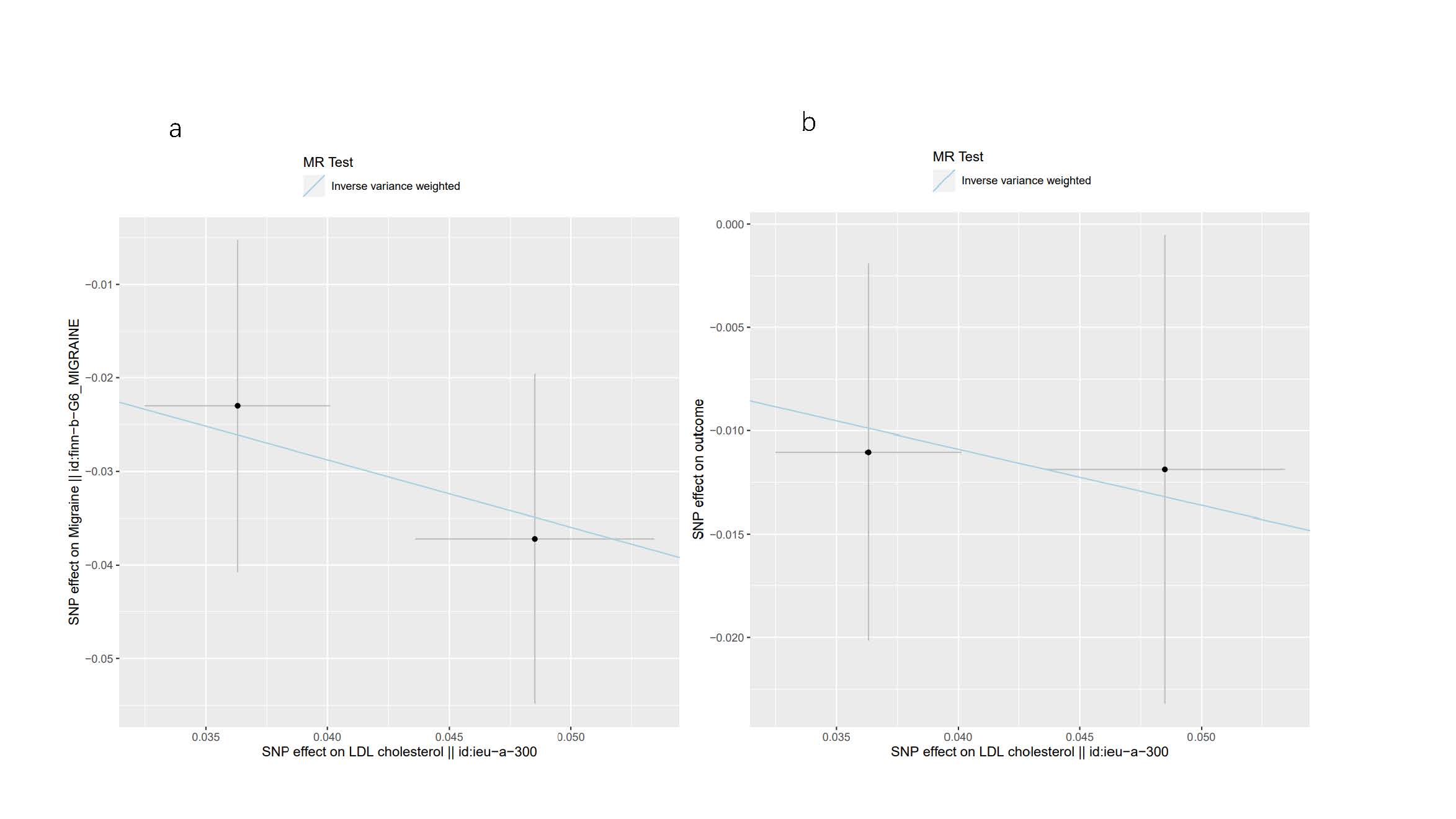

Supplement: Supplementary file 10 — Additional file 10: Figure S10. Scatter plot of the association between LDL and migraine using SNPs within or near the NPC1L1 locus in (a) Finngen dataset (b) Choquet dataset. [file 10194_2023_1633_MOESM10_ESM.jpg]

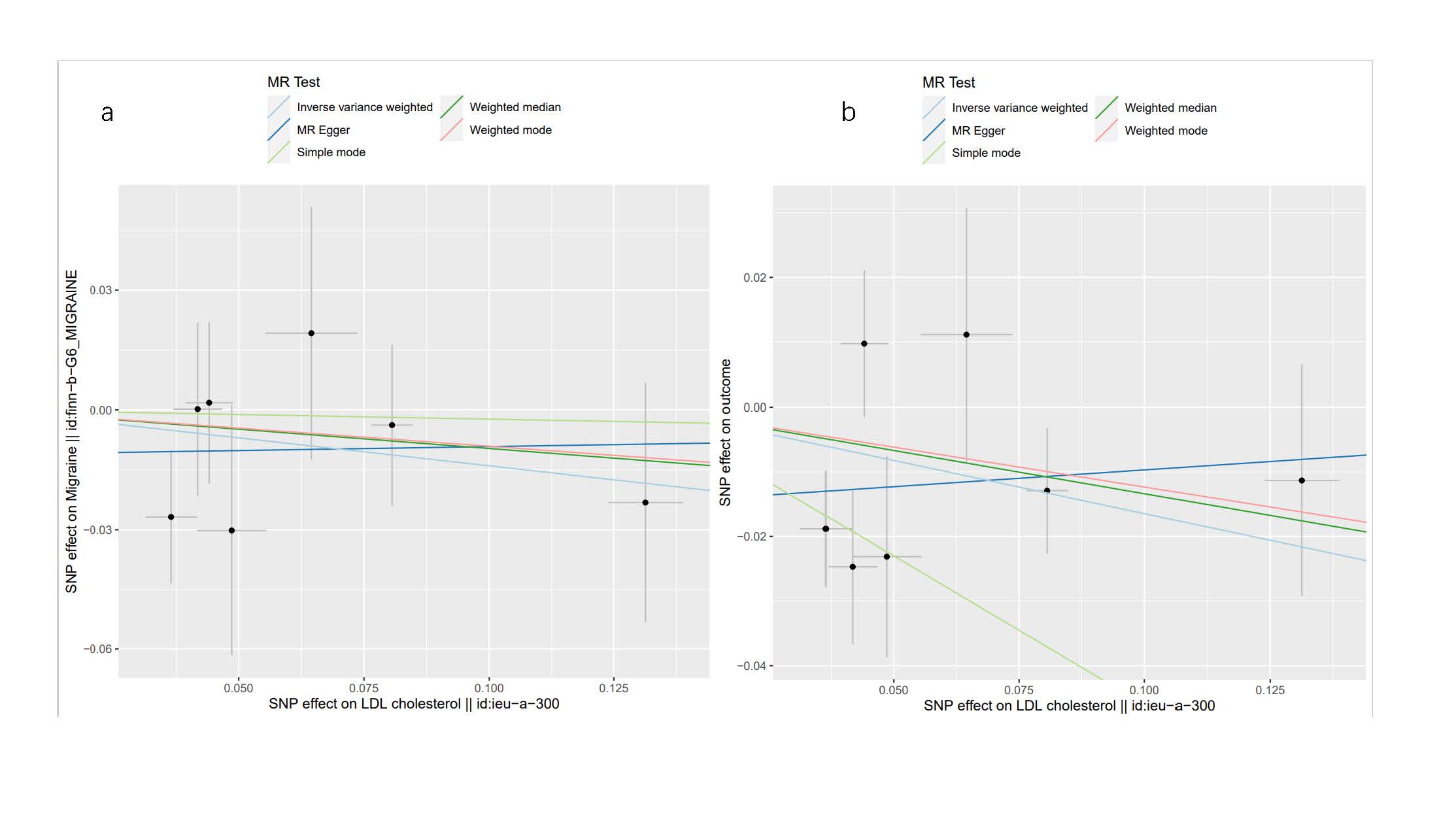

Supplement: Supplementary file 11 — Additional file 11: Figure S11. Scatter plot of the association between LDL and migraine using SNPs within or near the ABCG5/ABCG8 locus in (a) Finngen dataset (b) Choquet dataset. [file 10194_2023_1633_MOESM11_ESM.jpg]

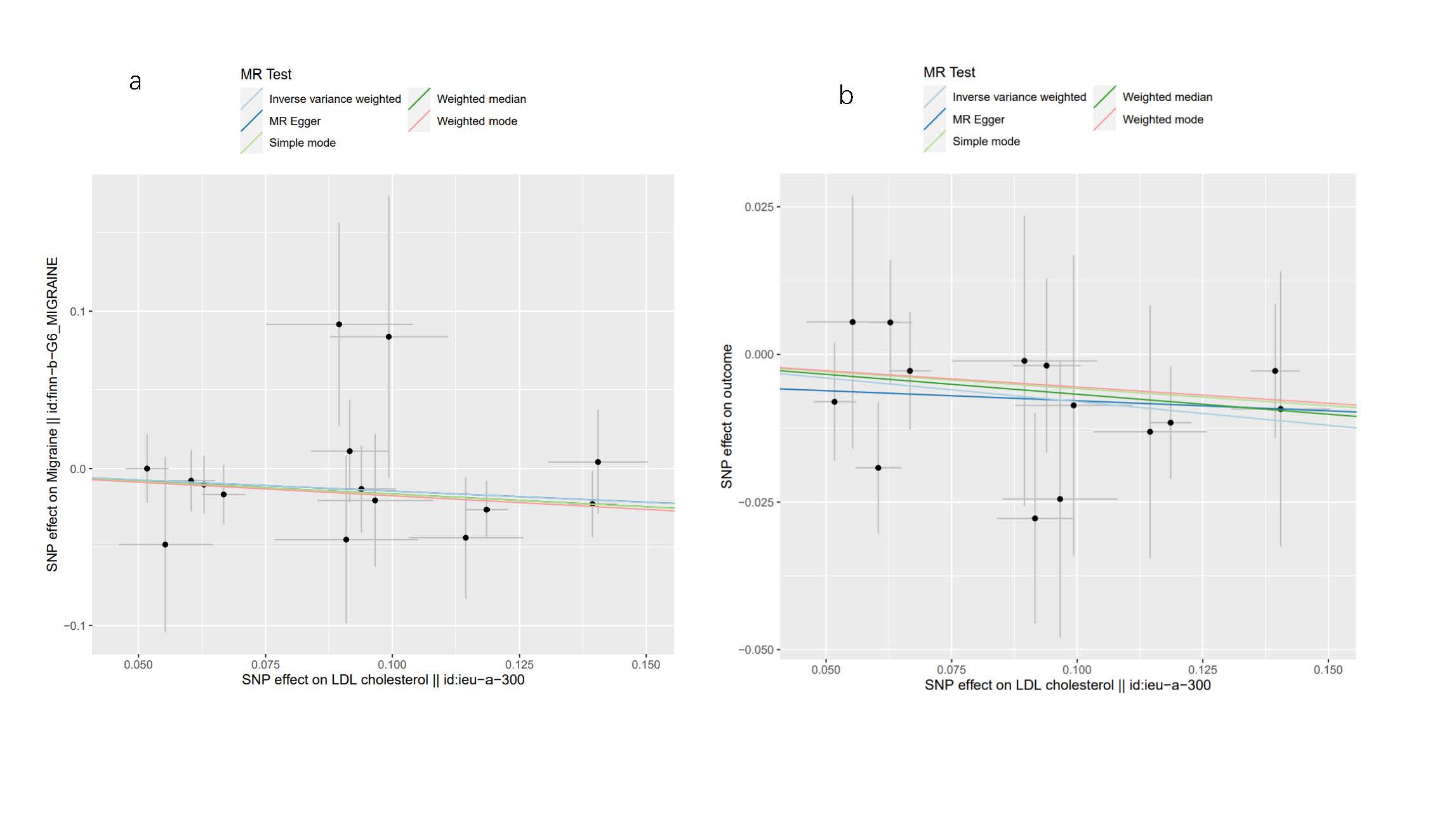

Supplement: Supplementary file 12 — Additional file 12: Figure S12. Scatter plot of the association between LDL and migraine using SNPs within or near the APOB locus in (a) Finngen dataset (b) Choquet dataset. [file 10194_2023_1633_MOESM12_ESM.jpg]

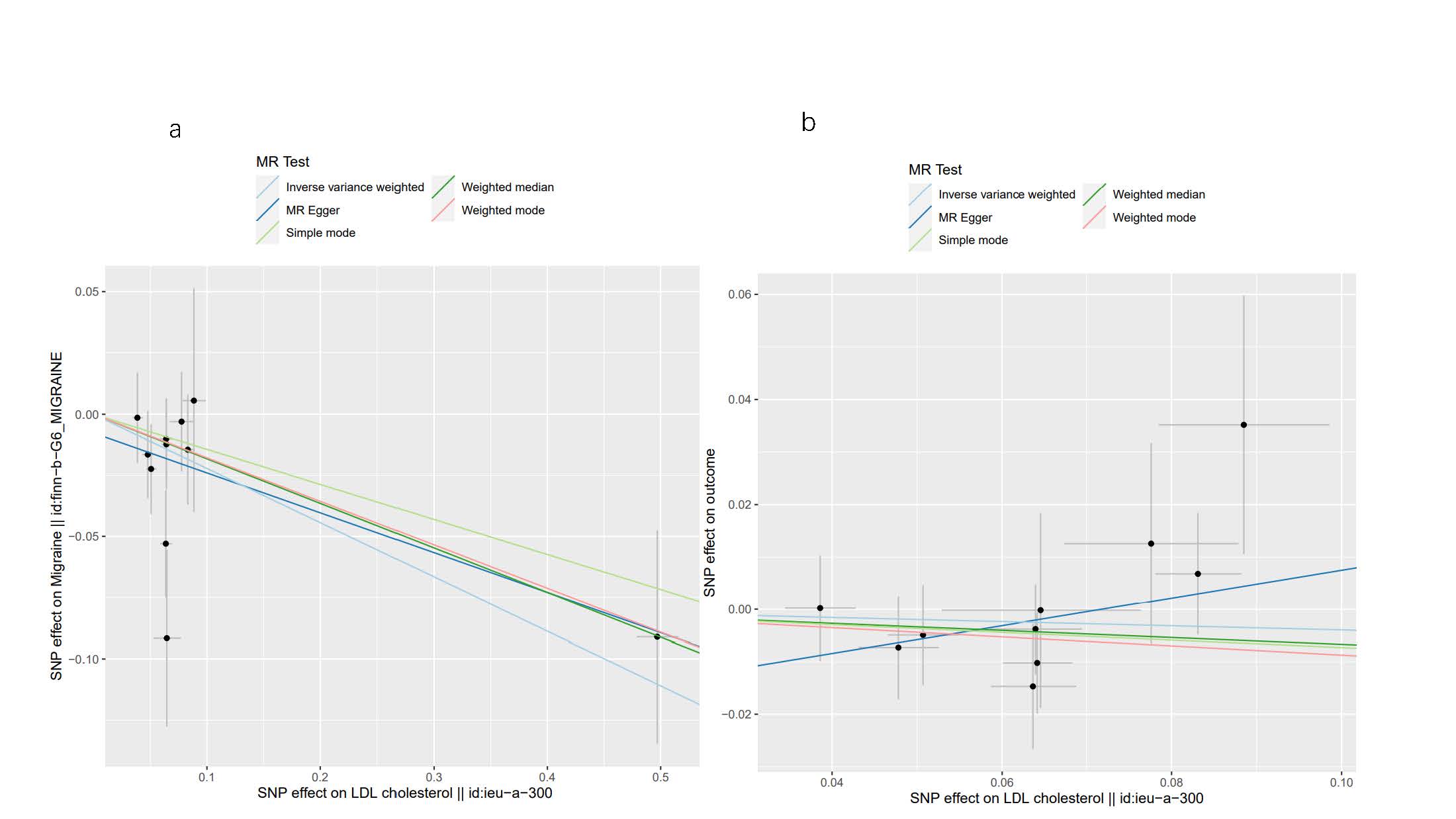

Supplement: Supplementary file 13 — Additional file 13: Figure S13. Scatter plot of the association between LDL and migraine using SNPs within or near the PCSK9 locus in (a) Finngen dataset (b) Choquet dataset. [file 10194_2023_1633_MOESM13_ESM.jpg]

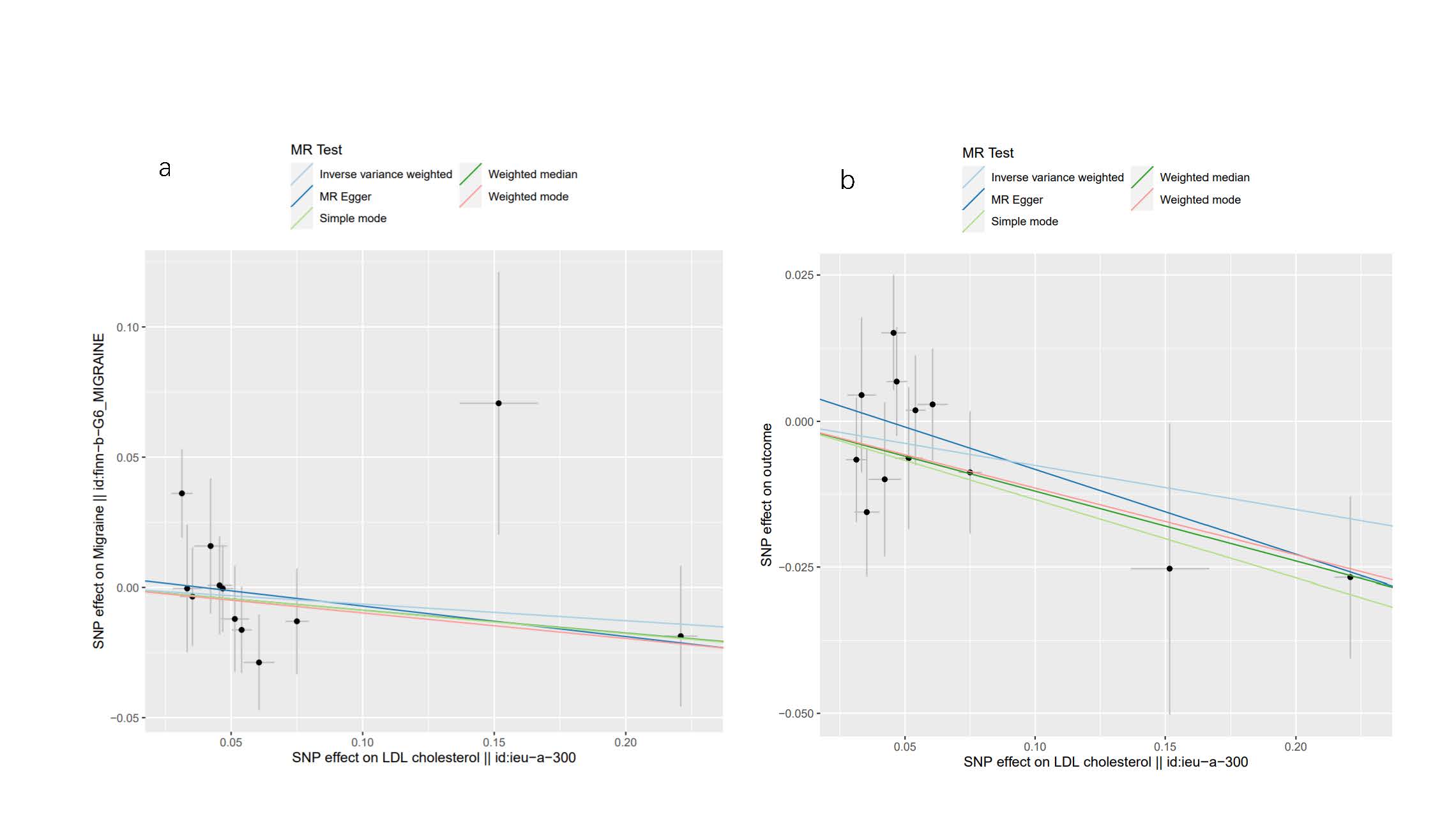

Supplement: Supplementary file 14 — Additional file 14: Figure S14. Scatter plot of the association between LDL and migraine using SNPs within or near the LDLR locus in (a) Finngen dataset (b) Choquet dataset. [file 10194_2023_1633_MOESM14_ESM.jpg]

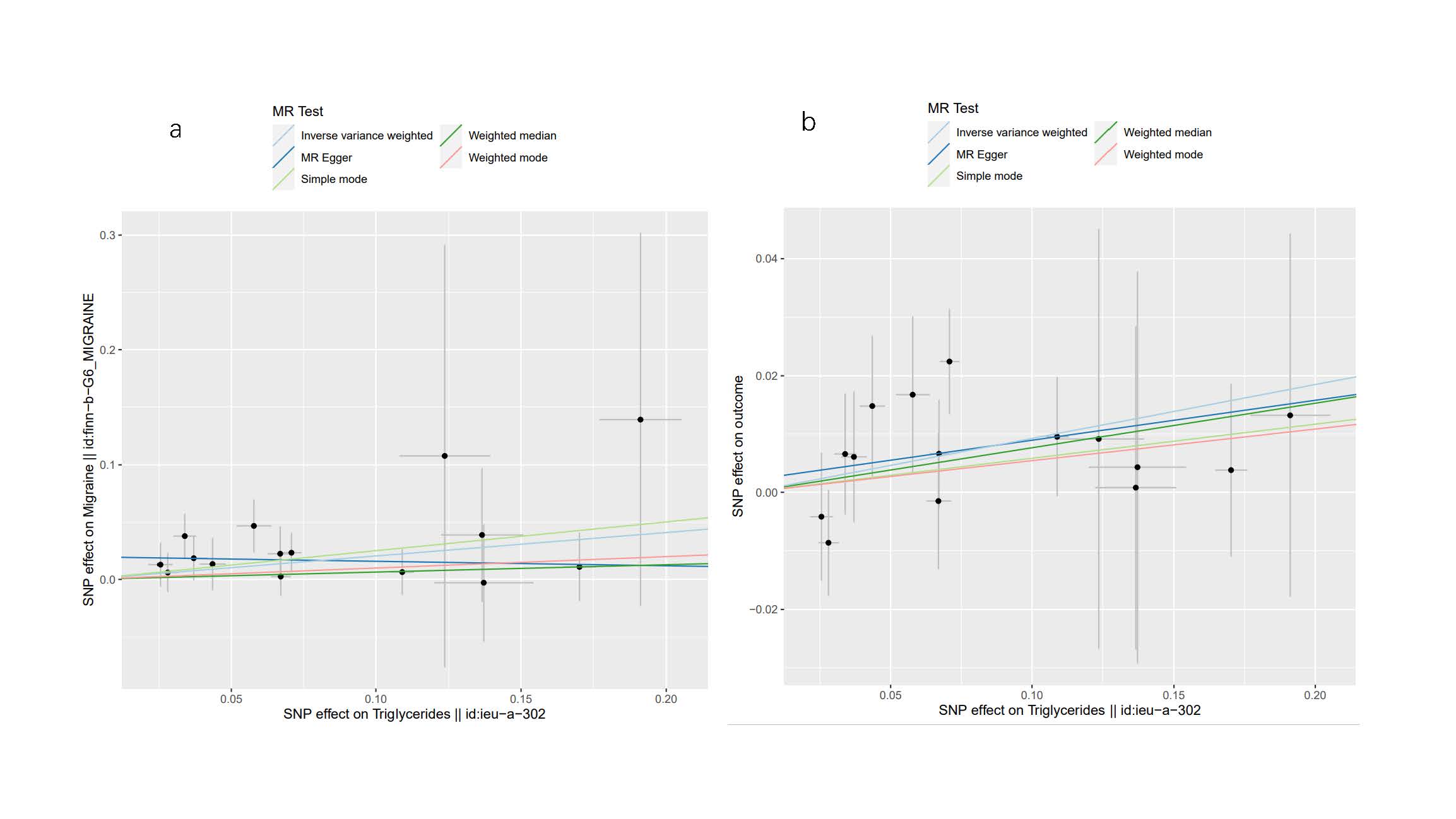

Supplement: Supplementary file 15 — Additional file 15: Figure S15. Scatter plot of the association between TG and migraine using SNPs within or near the LPL locus in (a) Finngen dataset (b) Choquet dataset. [file 10194_2023_1633_MOESM15_ESM.jpg]

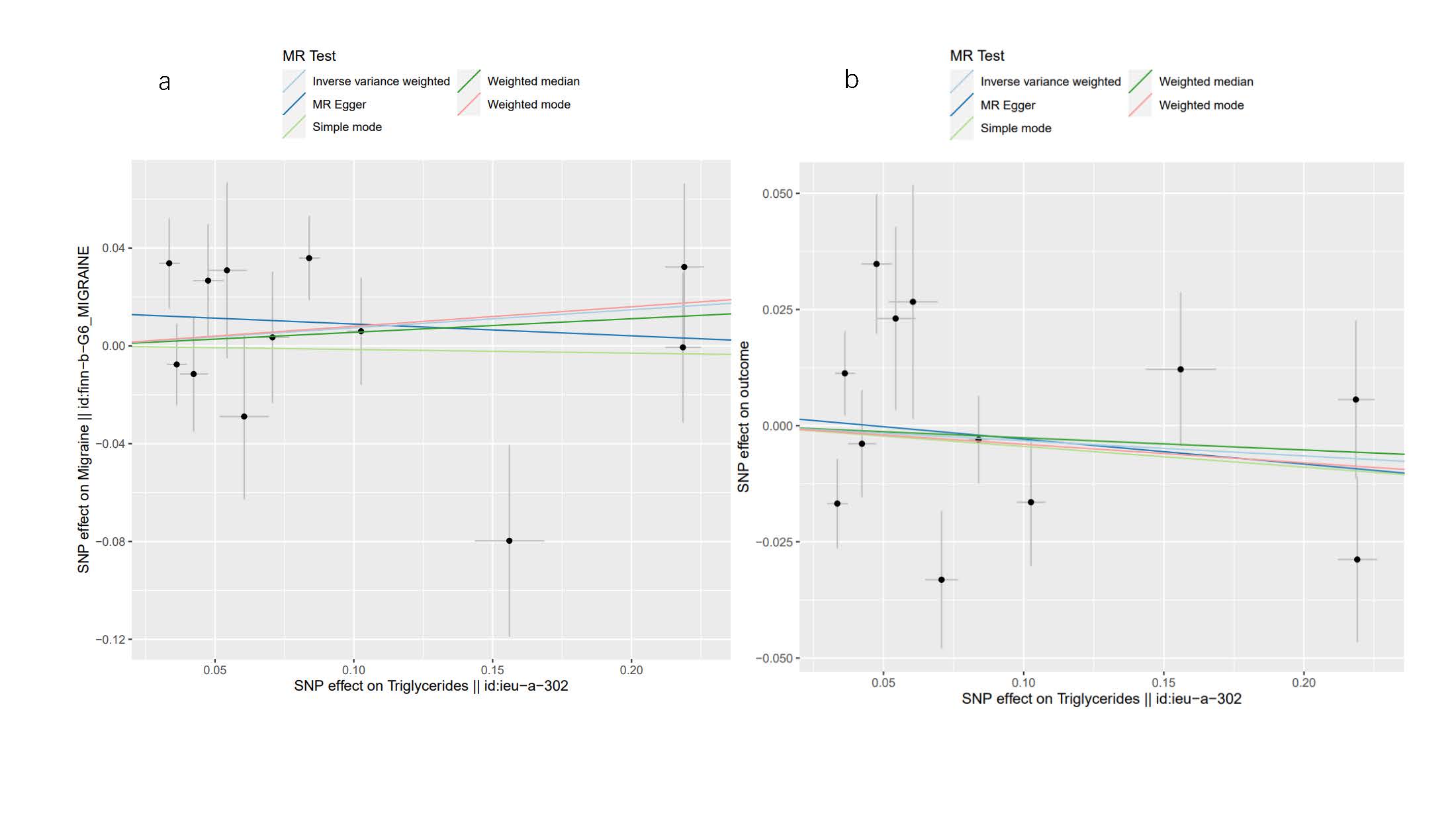

Supplement: Supplementary file 16 — Additional file 16: Figure S16. Scatter plot of the association between TG and migraine using SNPs within or near the APOC3 locus in (a) Finngen dataset (b) Choquet dataset. [file 10194_2023_1633_MOESM16_ESM.jpg]

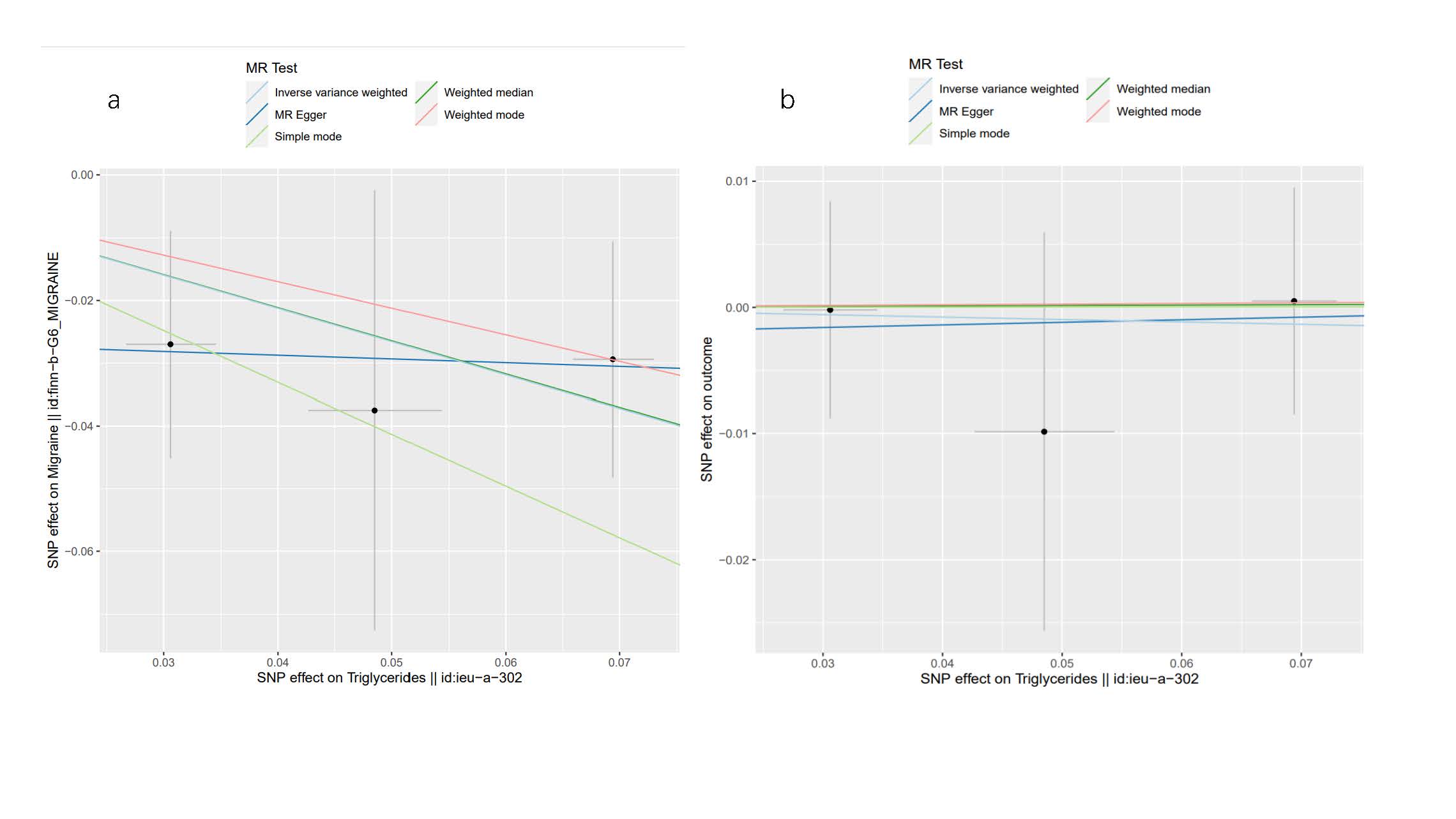

Supplement: Supplementary file 17 — Additional file 17: Figure S17. Scatter plot of the association between TG and migraine using SNPs within or near the ANGPTL3 locus in (a) Finngen dataset (b) Choquet dataset. [file 10194_2023_1633_MOESM17_ESM.jpg]

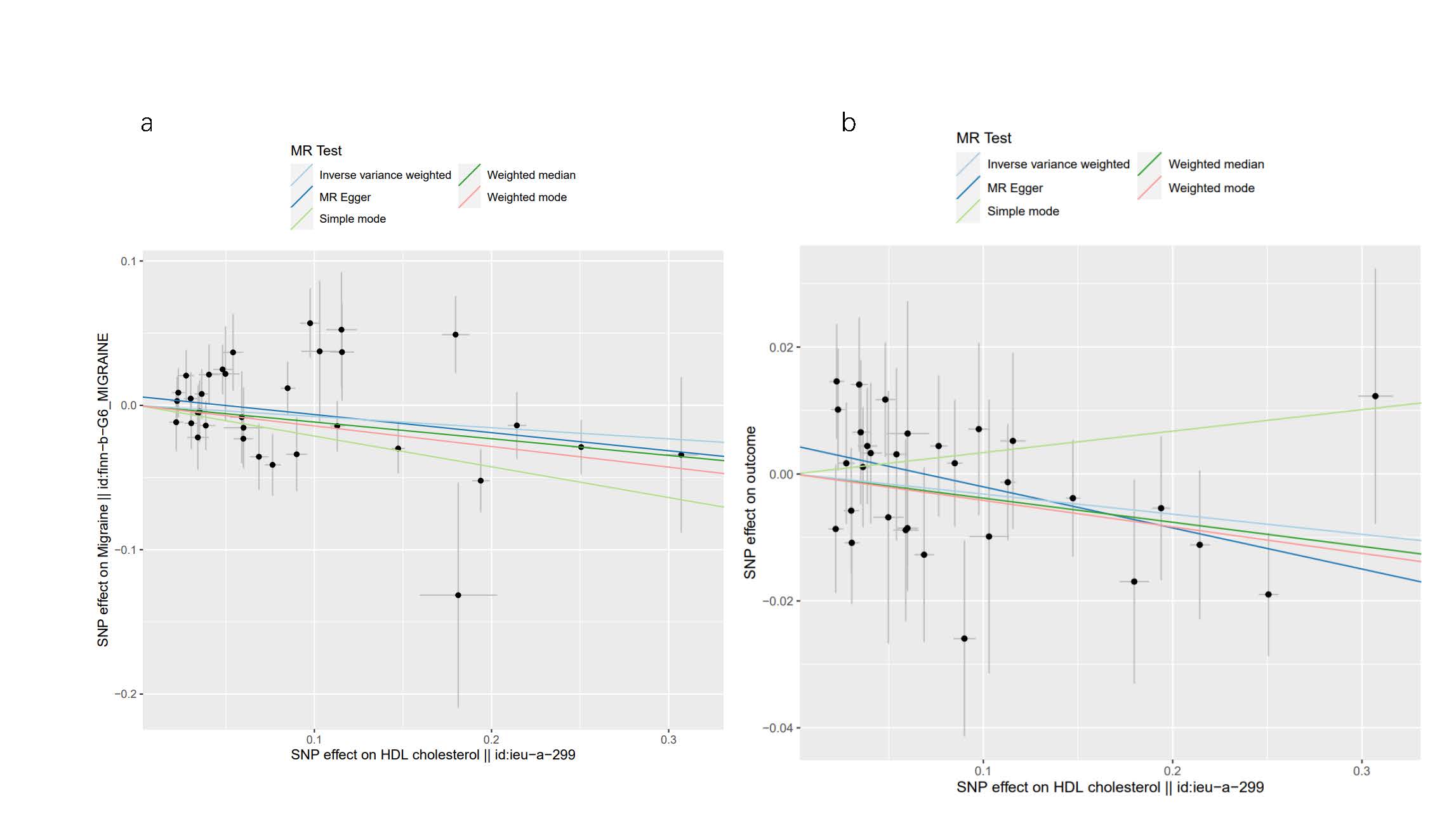

Supplement: Supplementary file 18 — Additional file 18: Figure S18. Scatter plot of the association between HDL and migraine using SNPs within or near the CETP locus in (a) Finngen dataset (b) Choquet dataset. [file 10194_2023_1633_MOESM18_ESM.jpg]
